# Cardiovascular progerin suppression and lamin A restoration rescues Hutchinson-Gilford progeria syndrome

**DOI:** 10.1161/CIRCULATIONAHA.121.055313

**Published:** 2021-10-25

**Authors:** Amanda Sánchez-López, Carla Espinós-Estévez, Cristina González-Gómez, Pilar Gonzalo, María J. Andrés-Manzano, Víctor Fanjul, Raquel Riquelme-Borja, Magda R. Hamczyk, Álvaro Macías, Lara del Campo, Emilio Camafeita, Jesús Vázquez, Anna Barkaway, Loïc Rolas, Sussan Nourshargh, Beatriz Dorado, Ignacio Benedicto, Vicente Andrés

**Affiliations:** 1 Centro Nacional de Investigaciones Cardiovasculares Carlos III (CNIC), Madrid, Spain; 2 Centro de Investigación Biomédica en Red de Enfermedades Cardiovasculares (CIBERCV), Spain; 3 Centre for Microvascular Research, William Harvey Research Institute, Barts and The London School of Medicine and Dentistry, Queen Mary University of London, London, UK

**Keywords:** Hutchinson-Gilford progeria syndrome, smooth muscle cell, cardiac myocyte

## Abstract

**Background:**

Hutchinson-Gilford progeria syndrome (HGPS) is a rare disorder characterized by premature aging and death mainly due to myocardial infarction, stroke, or heart failure. The disease is provoked by progerin, a variant of lamin A expressed in most differentiated cells. Patients look healthy at birth, and symptoms typically emerge in the first or second year of life. Assessing the reversibility of progerin-induced damage and the relative contribution of specific cell types is critical to determining the potential benefits of late treatment and to developing new therapies.

**Methods:**

We used CRISPR/Cas9 technology to generate *Lmna^HGPSrev/HGPSrev^* (*HGPSrev*) mice engineered to ubiquitously express progerin while lacking lamin A and allowing progerin suppression and lamin A restoration in a time- and cell-type-specific manner upon Cre recombinase activation. We characterized the phenotype of *HGPSrev* mice and crossed them with Cre transgenic lines to assess the effects of suppressing progerin and restoring lamin A ubiquitously at different disease stages as well as specifically in vascular smooth muscle cells (VSMCs) and cardiomyocytes.

**Results:**

Like HGPS patients, *HGPSrev* mice appear healthy at birth and progressively develop HGPS symptoms, including failure to thrive, lipodystrophy, VSMC loss, vascular fibrosis, electrocardiographic anomalies, and precocious death (median lifespan of 15 months versus 26 months in wild-type controls, p<0.0001). Ubiquitous progerin suppression and lamin A restoration significantly extended lifespan when induced in 6-month-old mildly symptomatic mice and even in severely ill animals aged 13 months, although the benefit was much more pronounced upon early intervention (84.5% lifespan extension in mildly symptomatic mice, p<0.0001, and 6.7% in severely ill mice, p<0.01). Remarkably, major vascular alterations were prevented and lifespan normalized in *HGPSrev* mice when progerin suppression and lamin A restoration were restricted to VSMCs and cardiomyocytes.

**Conclusions:**

*HGPSrev* mice constitute a new experimental model for advancing knowledge of HGPS. Our findings suggest that it is never too late to treat HGPS, although benefit is much more pronounced when progerin is targeted in mice with mild symptoms. Despite the broad expression pattern of progerin and its deleterious effects in many organs, restricting its suppression to VSMCs and cardiomyocytes is sufficient to prevent vascular disease and normalize lifespan.

## Non-standard Abbreviations and Acronyms

BSA: Bovine serum albumin
CNIC: Centro Nacional de Investigaciones Cardiovasculares
ECG: Electrocardiography
h: Hour(s)
H&E: Hematoxylin-eosin
HGPS: Hutchinson-Gilford progeria syndrome
*HGPSrev*: *Lmna^HGPSrev/HGPSrev^* mouse
HRP: Horseradish peroxidase
IP: Immunoprecipitation
LC-MS/MS: Liquid chromatography coupled to targeted tandem mass spectrometry
min: Minute(s)
MRI: Magnetic resonance imaging
NGS: Normal goat serum
OT: Off-target
o/n: Overnight
PRM: Precursor-reaction monitoring
RT: Room temperature
SD: Standard deviation
SEM: Standard error of the mean
SEMS: Spin-echo multi-slice
*SM22α-Cre*: B6.Cg-Tg(Tagln-cre)1Her/J mouse
SMA: Smooth muscle α-actin
TBST-T: Tris-buffered saline supplemented with 0.2% Tween 20
*Ubc-CreERT2^tg/+^*: B6.Cg *Ndor1^Tg(UBC-cre/ERT2)1Ejb^*/1J mouse
VSMC: Vascular smooth muscle cell
WT: Wild-type

## Introduction

HGPS is an ultra-rare genetic disorder (estimated prevalence 1 in 18-20 million people; https://www.progeriaresearch.org/) characterized by accelerated aging and premature death (average lifespan: 14.6 years)^1–3^. Most HGPS patients are heterozygous for a *de novo* synonymous mutation in the *LMNA* gene (c.1824C>T; p.G608G) that activates the use of a cryptic splice donor site in exon 11^4, 5^. In normal cells, *LMNA* expression mostly generates the alternatively-spliced isoforms lamin A and lamin C. The HGPS-causing mutation creates an aberrant *LMNA* mRNA that lacks 150 nucleotides in exon 11; this is translated into progerin, a permanently-farnesylated lamin A variant that exerts a dominant-negative effect^1^. Growth failure and alopecia manifest in HGPS patients as the first disease symptoms typically in the first or second year of life. Additional symptoms develop and worsen over time, including dermal and bone abnormalities, joint contractures, and loss of subcutaneous fat. The main medical problem in HGPS is severe cardiovascular disease, including generalized atherosclerosis and vascular calcification and stiffness, which ultimately provoke myocardial infarction, stroke, or heart failure, the causes of death in most HGPS patients^6, 7^. The US Food and Drug Administration recently approved the treatment of HGPS patients with lonafarnib (marketed as Zokinvy® by Eiger BioPharmaceuticals, US), a repurposed farnesyltransferase inhibitor that has extended the lifespan of HGPS patients by 2.5 years (17% increase)^8–10^. Nevertheless, there is a great need for better therapies to improve and eventually cure HGPS.

HGPS patients are typically diagnosed when symptoms are present or even severe, and treatment has historically been initiated at different disease stages^6^. It is therefore important to ascertain how late in life treatment can be initiated in symptomatic individuals while still yielding clinical benefit. Moreover, because progerin is expressed in most differentiated cells, it is vital to identify the cell types that would most benefit from treatment. To address these questions, here we generated the *LMNa^HGPSrev/HGPSrev^* (*HGPSrev*) mouse model, engineered to ubiquitously express progerin and lamin C and lack lamin A, while allowing progerin suppression and lamin A restoration upon Cre recombinase activation. We have characterized this model to assess the reversibility of progerin-induced damage by targeting progerin at early and late disease stages. Moreover, we have examined the consequences of suppressing progerin and restoring lamin A specifically in vascular smooth muscle cells (VSMCs) and cardiomyocytes, the major progerin targets.

## Methods

Additional methods are provided in the Supplemental Methods section online.

Data, analytical methods, and study materials will be made available to other researchers for the purposes of reproducing these results or replicating these procedures upon reasonable request directed to the authors’ laboratories.

### Study approval and mouse models

Mice used in this study were housed in the animal facilities at the Centro Nacional de Investigaciones Cardiovasculares (CNIC) under specific pathogen-free conditions at a constant temperature of 23°C, relative humidity 58%, and a 12-hour (h) dark/light cycle. Mouse health was monitored in a blinded manner at regular intervals throughout the study. Mouse handling and experimental procedures were performed to conform with current EU guidelines (Directive 2010/63/EU) and Recommendation 2007/526/EC regarding the protection of animals used for scientific purposes, enforced in Spanish law under Real Decreto 1201/2005; all procedures were approved by the Animal Protection Area of the Comunidad Autónoma de Madrid (PROEX 051/18) and the CNIC Ethics Review Board. To maximize information and minimize the number of animals used, we followed the 3Rs principles (Replace, Reduce and Refine)^11^ and the ARRIVE guidelines (Animal Research: Reporting of In Vivo Experiments)^12^ throughout this study.

Studies were carried out in C57BL/6J mice fed a regular rodent chow diet. Equal numbers of females and males were used. *Ubc-CreERT2^tg/+^* mice (B6.Cg *Ndor1^Tg(UBC-cre/ERT2)1Ejb^*/1J)^13^ and *Lmna^G609G/G609G^* mice^14^ were kindly provided by Dr. Mariano Barbacid and Dr. Carlos López-Otín, respectively. The generation of *HGPSrev* mice is explained below. *HGPSrev* mice were crossed with *Ubc-CreERT2^tg/+^* mice to generate *Lmna^HGPSrev/HGPSrev^ Ubc-CreERT2^tg/+^* mice with the Cre transgene in heterozygosis and exhibiting time-conditional Cre activity (referred to as *HGPSrev-Ubc-CreER^T2^* mice). *SM22α-Cre* mice (B6.Cg-Tg(Tagln-cre)1Her/J)^15^ (The Jackson Laboratory, Bar Harbor, ME USA) were crossed with *HGPSrev* mice to generate *Lmna^HGPSrev/HGPSrev^-SM22α-Cre* mice (*HGPSrev-SM22α-Cre* mice).

### Double stranded (dsDNA) donor template design for *Lmna^HGPSrev^* strain generation

**Figure 1:**
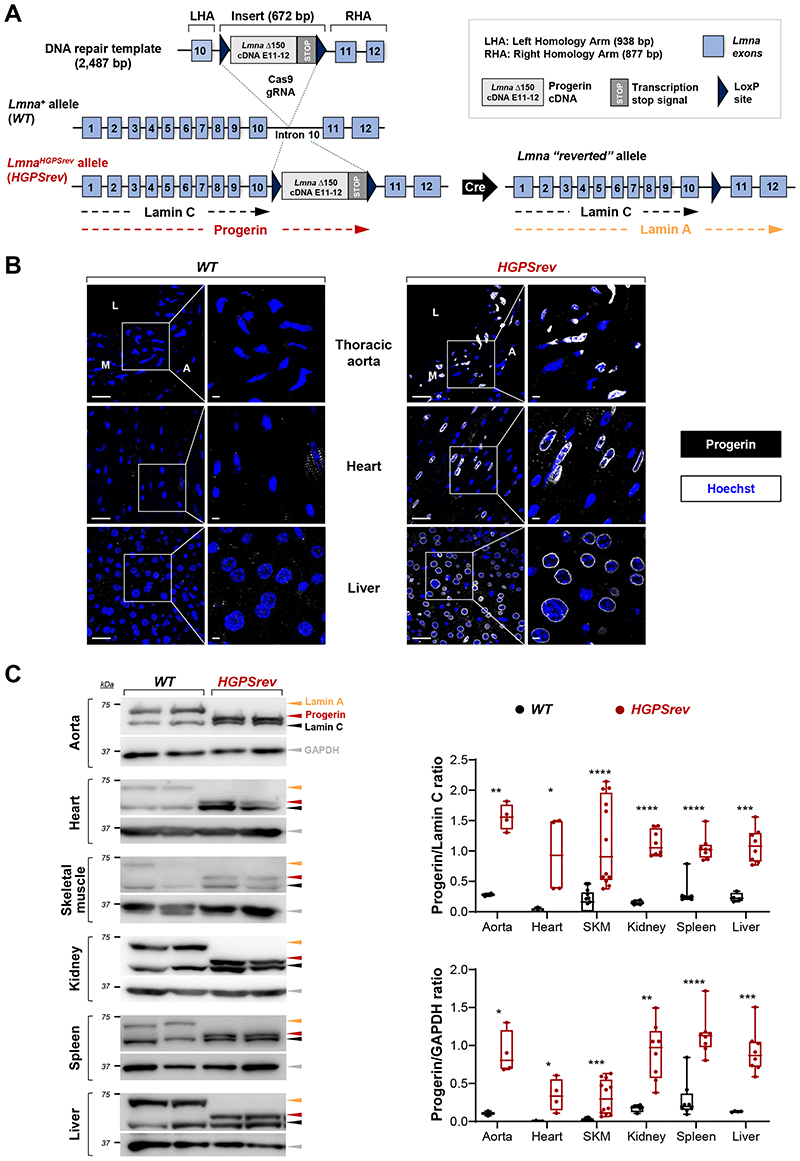
*Lmna^HGPSrev/HGPSrev^ (HGPSrev)* mice exhibit ubiquitous progerin expression and undetectable lamin A expression. **(A)** CRISPR-Cas9 strategy for generating *HGPSrev* mice (**see details in** Methods and Supplemental Fig. S1A). Cre activity generates a *Lmna* “reverted” allele that causes progerin suppression and lamin A restoration. **(B)** Representative immunofluorescence images showing progerin expression (white) and nuclei (blue) in *wild-type* and *HGPSrev* mice. Scale bar, 25 μm. **(C)** Western blot of lamin A/C, progerin and GAPDH in 2-month-old *WT* and *HGPSrev* mice. Six mice of each genotype were analyzed, and representative images are shown of two mice of each genotype. The graphs show the relative amount of progerin normalized using lamin C and GAPDH as controls. (n=3-13 *WT* mice; n=4-12 *HGPSrev* mice). Statistical analysis was performed by two-tailed t-test. *, p<0.05; **, p<0.01; ***, p<0.001; ****, p<0.0001. A, adventitia; L, lumen; M, media; SKM, skeletal muscle.

For homology directed repair, a 2,494 bp dsDNA donor template flanked by *EcoRI* and *Notl* recognition sites was synthesized (Figure 1A **in the Supplement**) and inserted into the pcDNA3.1 vector (Genscript; Piscataway, NJ USA). The dsDNA donor template contains a 938-bp left homology arm (*Lmna* intron 9, exon 10 and part of intron 10), a 672-bp insert harboring a loxP-flanked cDNA containing exons 11 and 12 from LmnaΔ150 (exon11Δ150 and the coding sequence of exon 12), followed by a bovine growth hormone polyadenylation transcriptional stop signal (BGH-polyA), and an 877-bp right homology arm (part of *Lmna* intron 10, exon 11, intron 11 and part of exon 12).

### Oocyte microinjection and implantation into pseudo-pregnant females

Hormonal superovulation was induced in 10 immature female mice (3-5 weeks old, C57BL/6J genetic background) by intraperitoneal hormone injection. Mice first received an injection of 0.1 mL (5 IU) of pregnant mare serum gonadotropin, followed 48 h later by an injection of 0.1 mL (5 international units) of human chorionic gonadotropin. Immediately after the second injection, animals were mated with appropriate stud males. One day after mating, females were checked for vaginal plugs, and those with a positive result were sacrificed. Oviducts from sacrificed females were extracted and transferred to M2 culture medium (M7167; Sigma-Aldrich, St Louis, MO USA) containing 350 μg/mL hyaluronidase (H3884, Sigma-Aldrich). Each ampulla was localized and opened to release the cumulus mass, and oocytes were separated after incubation at 37 ºC for 1-2 minutes (min) and then transferred to fresh M2 medium for washing. Zygotes were incubated in Evolve-KSOM culture medium (ZEKS-050; Zenith Biotech, Cork, Ireland) at 37 ºC in a 5% CO_2_/5% O_2_ atmosphere, until they were ready for pronuclear microinjection^16^. Zygotes were microinjected with 1-2 pL of a microinjection solution containing guide RNAs, dsDNA donor template and Cas9 endonuclease (Table 1 **in the Supplement**). Zygotes were incubated overnight (o/n) at 37 ºC and 5% CO_2_/5% O_2_ in Evolve-KSOM medium (ZEKS-050; Zenith Biotech) to reach the 2-cell stage^16^. Embryos at the 2-cell stage were transferred to pseudo-pregnant female mice by passing a sterile glass needle through the infundibulum. Three weeks later, 34 pups were weaned from their gestational mothers.

### Identification of founder *HGPSrev* mice

To identify mice carrying the *Lmna^HGPSrev^* allele, we extracted genomic DNA from the tails of the 34 mouse pups following a standard protocol with Proteinase K (EO0492; Thermo Fisher, Waltham, MA USA) and performed PCR with specific primers (Table 2 **in the Supplement - Founders: PCR-1**). Additional PCR reactions were run to identify mice carrying a single copy of the mutant allele at the proper location in the *Lmna* locus (Table 2 **in the Supplement - Founders: PCR-2 and PCR-3**). Genomic DNA from the 4 pups carrying a single copy of the edited allele was amplified by PCR and sequenced at the Sequencing Service of the Centro Nacional de Investigaciones Oncológicas (CNIO, Madrid, Spain) (Figure 1B **in the Supplement**).

### Genotyping of *HGPSrev* mice

To genotype *HGPSrev, HGPSrev-Ubc-CreERT2* and *HGPSrev-SM22α-Cre* mice, genomic DNA was extracted from the tail following a standard Proteinase K protocol (EO0492; Thermo Fisher) and PCR reactions were performed with specific primers (Table 2 **in the Supplement - Genotyping**).

### Isolation, immortalization and transfection of mouse embryonic fibroblasts (MEFs)

Embryos isolated at embryonic day 13.5 were minced and incubated for 20 min in 2X trypsin-EDTA (0.5% Trypsin, 0.53 mM EDTA•4Na) (15400-054; Invitrogen, Carlsbad, CA USA). MEFs from each embryo were plated separately and incubated at 37 °C in complete growth medium (DMEM supplemented with 10% heat-inactivated fetal bovine serum, 5% non-essential amino acids, 5% penicillin/streptomycin, and 5% L-glutamine (v/v)). To generate virus to immortalize MEFs, HEK293T cells were seeded and transfected with pCL-Puro-SV40 LT retroviral vector (13970; Addgene, Watertown, MA USA) and pCL-ECO retroviral packaging plasmid (kindly provided by Dr. Manuel Serrano) using Fugene 6 (E2692; Promega, Madison, WI USA). Supernatants containing retroviral particles were harvested every 12 h over 2 days, filtered through 0.45 μm pores, mixed with polybrene (8 μg/mL) (9268-5G; Sigma-Aldrich), and used to infect 2.5-5x10^5^ MEFs in 100 mm dishes. Infected MEFs were serially passaged to select immortalized cells by adding 2 μg/mL puromycin (P8833, Sigma-Aldrich).

Immortalized MEFs were seeded at ≈80% confluence and were transfected with 1.5 μg pPB CAG ER-Cre-ER IRES Zeocin^17^ and 4.5 μg pCMV-hyPBase^18^ mixed with 18 μL TransIT-LT1 Transfection Reagent (MIR2300; GeneFlow, Staffordshire, UK) in DMEM. Zeocin-resistant cells were selected by incubation for at least 10 days in the presence of 400 μg/mL zeocin (R25005; Thermo Fisher).

### Longevity studies

Starting at 4-8 weeks of age, mice were weighed and inspected for health and survival twice a week. Health status was examined by a blinded veterinarian. Animals that met humane end-point criteria were euthanized and the deaths recorded for the survival curve analysis. The robust regression and outlier removal (ROUT) test was performed in all survival experiments, and identified two outliers: a tamoxifen-injected *HGPSrev-Ubc-CreER^T2^* mouse that died at 34 months and an oil-injected mouse that died at 17 months (in both cases, administration of tamoxifen/oil started at ≈13 months). These mice were excluded from the survival curve and statistical analysis.

### Histology and immunofluorescence

Mouse tissues were fixed in 4% formaldehyde solution (prepared from paraformaldehyde) for 24 h at 4 ºC, dehydrated through an ascending series of ethanol concentrations, and finally embedded in paraffin and cut into serial 4-μm sections.

For immunofluorescence studies, tissue cross-sections were deparaffinized, rehydrated and washed in PBS. Antigen retrieval was performed by boiling the sections in 10 mM sodium citrate buffer (pH 6) for 20 min, and samples were then blocked and permeabilized for 1 h at room temperature (RT) in PBS supplemented with 0.3% Triton X-100, 5% BSA, and 5% normal goat serum (NGS) (005-000-001, Jackson ImmunoResearch, West Grove, PA USA). Heart samples were additionally blocked with 100 mM glycine (5001901000; Merck, Kenilworth, NJ USA). Primary and secondary antibodies were diluted in PBS supplemented with 0.3% Triton X-100, 5% BSA, and 2.5% NGS. Primary antibodies included rat monoclonal anti-CD31 (1:50, DIA-310; Dianova, Hamburg, Germany) and rabbit polyclonal anti-progerin (1:800, generated by the Nourshargh laboratory using peptide immunogens and standard immunization procedures). After overnight incubation at 4°C, samples were washed and then incubated with the appropriate fluorescently-labeled secondary antibodies for 2 h at RT (1:400 goat anti-rat Alexa Fluor 488 A-11006 or 1:400 goat anti-rabbit Alexa Fluor 647 A-21245; Invitrogen) together with anti-α-smooth muscle actin-Cy3 (1:300, C6198; Sigma-Aldrich) and Hoechst nuclear stain (bisBenzimide H 33342 trihydrochloride, B2261; Sigma Aldrich). Samples were washed and then mounted in Fluoromount-G imaging medium (4958-02; eBioscience, San Diego, CA USA). Images were acquired with a Leica SP5 DMI 6000B (Leica® Microsystems, Wetzlar, Germany) or a LSM 700 Carl Zeiss (Zeiss, Oberkochen, Germany) confocal microscope.

For staining with hematoxylin-eosin (H&E) and Masson’s trichrome, tissue cross-sections were deparaffinized, rehydrated and washed in PBS. Stained sections were scanned with a NanoZoomer-RS scanner (Hamamatsu, Japan), and images were exported with NDP.view2 software (Hamamatsu) and quantified with user-customized macros in Fiji software by an operator blinded to genotype. The thickness of the subcutaneous fat layer was qualitatively scored from 1 (thinnest) to 5 (thickest) by five independent observers and mean results are presented for each mouse. For quantitative analysis, regions containing epidermis, hypodermis, and muscle layers were examined by a single blinded manner observer, and the mean thickness was calculated from 10 independent measurements per mouse.

### mRNA isolation and reverse transcription for PCR detection of lamin A and progerin

Total RNA was extracted from powdered mouse tissue samples using TriReagent Solution (AM9738, Thermo Fisher) and processed by alcohol precipitation. RNA was quantified in a NanoDrop ND-1000 spectrophotometer and 1-2 μg were transcribed to cDNA using the High-Capacity cDNA Reverse Transcription Kit (4368814; Applied Biosystems, Foster City, CA USA). Lamin A and progerin mRNAs were detected as previously described^19^. cDNA samples (100 ng) were amplified by PCR, and products were separated on a 1.5% agarose gel. Images were acquired with the Molecular Imager® Gel Doc™ XR+ System (Bio-Rad).

### Plasma biochemistry

Blood was extracted from the mandibular sinus of live mice or from the heart (by cardiac puncture) or infrarenal abdominal aorta of euthanized animals. For biochemical analysis, plasma was obtained from blood samples collected in Microvette EDTA tubes (Sarstedt, Newton, NC USA) by specialized CNIC Animal Facility staff and centrifuged at 180-200 g for 15 min at 4°C. Biochemical variables were analyzed using a Dimension RxL Max Integrated Chemistry System (Siemens Healthineers, Erlangen, Germany).

### Electrocardiography (ECG)

Mice were anesthetized with 1.5-2% isoflurane, and 4 ECG electrodes were inserted subcutaneously into the limbs. ECG was recorded in the morning for approximately 2 min using a MP36R data acquisition workstation (Biopac Systems, Goleta, CA, USA). ECG data were exported with AcqKnowledge software (Biopac Systems) and automatically analyzed using custom R scripts developed to: 1) remove noise and baseline fluctuations; 2) detect heart beats, peaks and waves; 3) exclude artifacts; and 4) calculate QT intervals and T-wave steepness.

### Tamoxifen administration

Tamoxifen (4-hydroxy-tamoxifen, H6278; Sigma Aldrich) was dissolved in ethanol for cell studies or in corn oil (C8267; Sigma Aldrich) for mouse studies. The corn-oil preparation was incubated at 55°C until the tamoxifen was fully dissolved and then passed through a 0.22 μm filter.

Zeocin-resistant *WT* and *HGPSrev* MEFs were exposed to 25 nM tamoxifen and protein lysates were prepared 24, 48 and 72 h after tamoxifen administration. Negative controls were treated with equal volume of vehicle (ethanol) for 72 h.

Mice were randomized to tamoxifen or oil groups balanced for age and sex. For proof-of-concept studies, ≈3-month-old *HGPSrev-Ubc-CreER^T2^* mice received daily intraperitoneal oil or tamoxifen injections (2 mg/day/mouse) for 10 days; the mice were sacrificed one week after finishing the treatment and tissues were extracted for western blot analysis. For the analysis of *HGPSrev-Ubc-CreER^T2^* mice at different disease stages, tamoxifen administration was commenced at different ages. For longitudinal studies of survival, health status and body-weight evolution, the effect on early disease was assessed by starting oil/tamoxifen administration at ≈6 months of age, coinciding with the beginning of growth failure, whereas late disease was assessed by starting treatment at ≈13 months of age, when mice had developed severe symptoms and were close to maximum survival (Table 1). For ECG and histopathological analysis, the effect on intermediate disease (characterized by mild cardiovascular symptoms) was assessed by starting treatment at ≈9 months of age. All mice received daily intraperitoneal injections (1 mg/day/mouse) over 5 days and were sacrificed at 14.5 months of age, at which point 4 out of 10 oil-injected animals had died and only 1 out of 12 tamoxifen-injected animals had died.

### Statistical analysis

Quantitative data are presented as the mean ± the standard error of the mean (SEM) unless otherwise stated. Statistical tests were applied after the determination of normal distribution (Shapiro-Wilk normality test) and equality of variances (F test). In experiments with two groups and normal distribution, the statistical significance of differences was assessed by unpaired two-tailed Student’s t-test. For non-normally distributed data in experiments with two groups, we used the Mann-Whitney test. In experiments with more than two groups of normally distributed populations, we applied one-way ANOVA followed by the post hoc Tukey test. For non-normally distributed data, the non-parametric equivalent Kruskal-Wallis test was performed. When more than 2 groups were assessed over time without sphericity, we used mixed-effects analysis (two-way ANOVA) with the Geisser-Greenhouse correction and Sidák’s multiple comparisons. Kaplan-Meier survival curves were compared by the log-rank (Mantel-Cox) test. Data on body-weight evolution were analyzed by unpaired multiple t-tests with the Holm-Sidák correction.

All statistical tests were run in GraphPad Prism® 9.0.0. Differences were considered statistically significant when p-values were below 0.05: *, p<0.05; **, p<0.01; ***, p<0.001; ****, p<0.0001.

## Results

### *HGPSrev* mice develop progeroid symptoms and allow Cre-dependent progerin suppression and lamin A restoration

**Figure 2:**
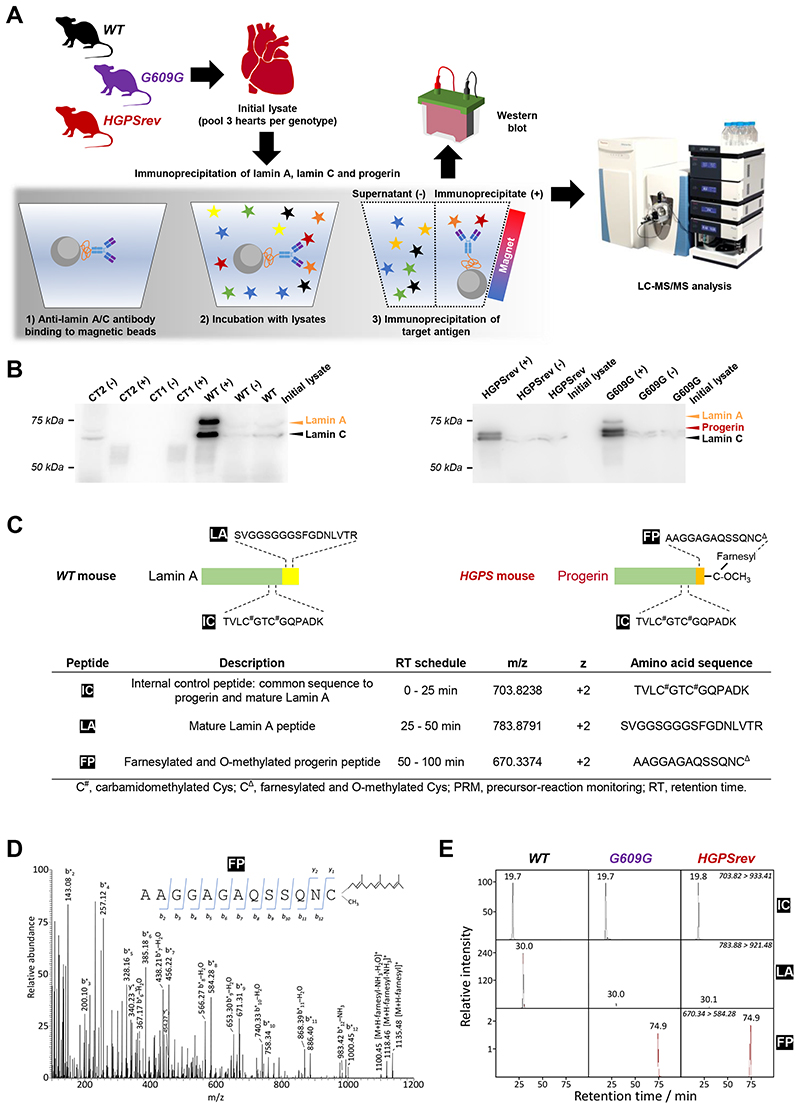
Targeted precursor-reaction monitoring (PRM) analysis to examine progerin farnesylation in mouse heart lysates. **(A)** Workflow for the LC-MS/MS analysis of proteins extracted from mouse hearts and immunoprecipitated with anti-lamin A/C antibodies that recognize lamin A, lamin C, and progerin. For each genotype, each sample was the pool of 3 hearts. *WT, wild-type* mice; *G609G, Lmna^G609G/G609G^* mice*; HGPSrev, Lmna^HGPSrev/HGPSrev^* mice. **(B)** Western blots using anti-lamin A/C antibody to check the enrichment of lamin A, lamin C, and progerin in the immunoprecipitated material and supernatant (+, immunoprecipitated; -, supernatant). Controls included samples containing only beads and antibody (CT1) and only beads and protein extract (CT2). A 10 μL aliquot of each sample was loaded onto the gel; see details in Supplemental Methods. **(C)** Surrogate peptides used to detect mature lamin A and progerin: *IC*, internal control peptide (present in both lamin A and progerin); *LA*, lamin A peptide (specific for lamin A); *FP*, farnesylated progerin peptide (specific for progerin). **(D)** MS^2^ fragmentation spectrum from *FP* obtained in the PRM assay. The insert shows ion ascription to the main fragment-ion series (C-terminal y-series and N-terminal b-series). **(E)** MS/MS (tandem mass spectrometry) extracted ion chromatograms of *IC*, *LA*, and *FP* peptides obtained from the time-scheduled PRM assay for the detection of lamin A and progerin. The ion traces were obtained using fragment ion y^+^_9_ from *IC*, y^+^_8_ from *LA*, and b^+^_8_ from *FP*.

We used the CRISPR-Cas9 strategy to generate the *HGPSrev* mouse model, engineered to ubiquitously express progerin and lamin C and to lack lamin A, while allowing progerin suppression and lamin A restoration upon Cre recombinase activation (Figure 1A, **and** Figure 1A,B **in the Supplement**). Western blot analysis in protein lysates from the tails of founder *HGPSrev* mice revealed progerin and lamin C expression and undetectable lamin A (Figure 1C **in the Supplement**). *HGPSrev* mice obtained after breeding founder mice expressed progerin in all tissues tested, as assessed by reverse transcriptase-PCR (Figure 1D **in the Supplement**: compare *wild-type* (*WT*) with *HGPSrev*), immunofluorescence (Figure 1B, **and** Figure 2 **in the Supplement**: compare *WT* with *HGPSrev*), and western blot (Figure 1C). Analysis of multiple tissues by semiquantitative PCR showed lower progerin mRNA content in *HGPSrev* mice than in *Lmna^G609G/G609G^* mice, a widely used HGPS model^14^ (Figure 1D **in the Supplement**: compare *HGPSrev* with *G609G*).

We next examined progerin farnesylation in *HGPSrev* and *Lmna^G609G/G609G^* mice, using *WT* mice as a negative control. Proteins were immunoprecipitated from heart lysates with an anti-lamin A/C antibody that recognizes lamin A/C and progerin, and samples were analyzed by western blot and liquid chromatography coupled to targeted tandem mass spectrometry (LC-MS/MS) using a high-resolution precursor-reaction monitoring (PRM) method (Figure 2A **and** Supplemental Methods). Compared with the initial lysates, *WT* heart immunoprecipitates were enriched in lamin A/C (Figure 2B, *WT* (+) versus *WT* Initial lysate), *HGPSrev* heart immunoprecipitates were enriched in progerin and lamin C and contained no detectable lamin A (Figure 2B, *HGPSrev (+)* versus *HGPSrev* Initial lysate), and *Lmna^G609G/G609G^* heart immunoprecipitates were enriched in progerin and lamin A/C (Figure 2B, *G9609G (+)* versus *G609G* Initial lysate). For PRM, we monitored a peptide common to lamin A and progerin (*IC, internal control*) and peptides specific for lamin A (*LA*) and farnesylated progerin (*FP*) (Figure 2C). The MS/MS spectra confirmed the presence of the farnesyl moiety in the monitored *FP* peptide (Figure 2D). MS/MS (tandem mass spectrometry) extracted ion chromatograms obtained from the time-scheduled PRM assay showed similar amounts of *FP* in *HGPSrev* and *Lmna^G609G/G609G^* hearts relative to the total amount of A-type lamin isoforms in these mutant strains, whereas *FP* was undetectable in *WT* hearts (Figure 2E). Consistent with this finding, *LA* was strongly depleted in *HGPSrev* and *Lmna^G609G/G609G^* hearts relative to WT hearts (Figure 2E).

**Figure 3:**
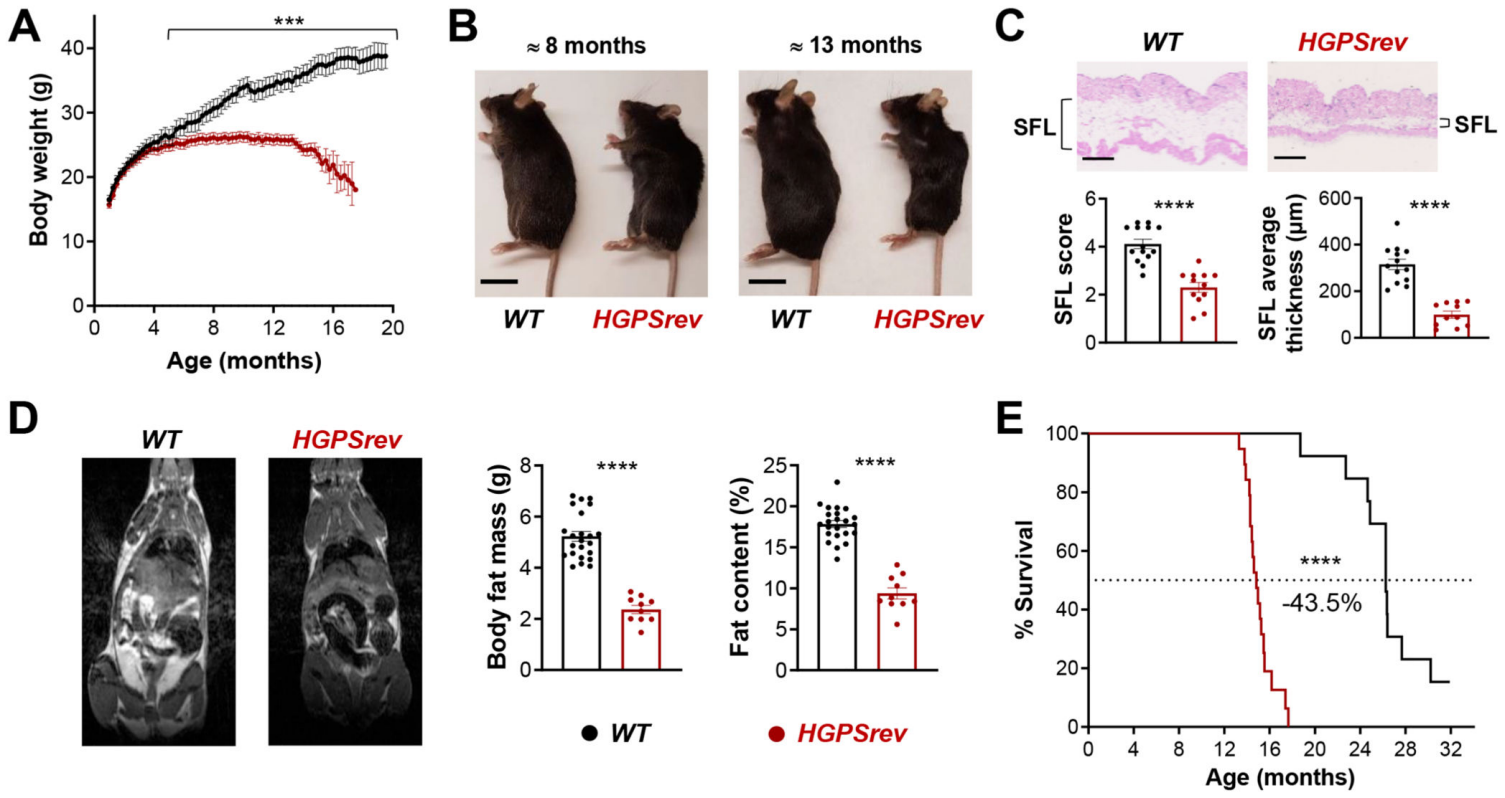
Progeroid phenotype in *Lmna^HGPSrev/HGPSrev^ (HGPSrev*) mice with ubiquitous progerin expression. **(A)** Postnatal body-weight curves (n=14 *WT*; n=22 *HGPSrev*). Differences were analyzed by unpaired multiple t-tests and the Holm-Sidák correction. **(B)** Representative images of ≈8- and ≈13-month-old mice. Scale bar, 2 cm. **(C)** Representative images of hematoxylin & eosin-stained skin from ≈13-month-old mice and the results of subcutaneous fat layer (SFL) score and thickness quantification (see Methods) (n=13-14 *WT*; n=11-12 *HGPSrev*). Statistical analysis was performed by two-tailed t-test. Scale bar, 500 μm. **(D)** Representative images of sagittal whole-body cross-sections obtained by MRI (fat shown in white) and quantification of body-fat mass and percentage fat content in ≈13-month-old mice (n=23 *WT*; n=10 *HGPSrev*). Differences were analyzed by two-tailed t-test. **(E)** Kaplan-Meier survival curve (n=13 *WT*; n=22 *HGPSrev*). Differences were analyzed by the Mantel-Cox test. ***, p<0.001; ****, p<0.0001. Data are mean±SEM. Each symbol represents one animal.

To examine potential off-target (OT) effects in *HGPSrev* mice, we used the online Off-Spotter tool (https://cm.jefferson.edu/Off-Spotter/). Compared with the 20 mer single guide RNA used for CRISPR/Cas9-dependent editing, this analysis identified 184 mouse genomic sequences containing 3, 4, or 5 mismatches (2, 16, and 166 sequences, respectively) (Figure 3A **in the Supplement**). We selected 3-mismatch sequences (OT-1 and OT-2) and 6 of the 4-mismatch sequences (OT-3, OT-4, OT-5, OT-6, OT-7, and OT-8) (Figure 3B **in the Supplement**), which were amplified by PCR using as template genomic DNA of *WT* and *HGPSrev* mice (n=5 per genotype). The PCR products had identical DNA sequence in all mice for all genomic regions examined (Figure 3C **in the Supplement**), indicating the absence of off-target effects.

**Figure 4:**
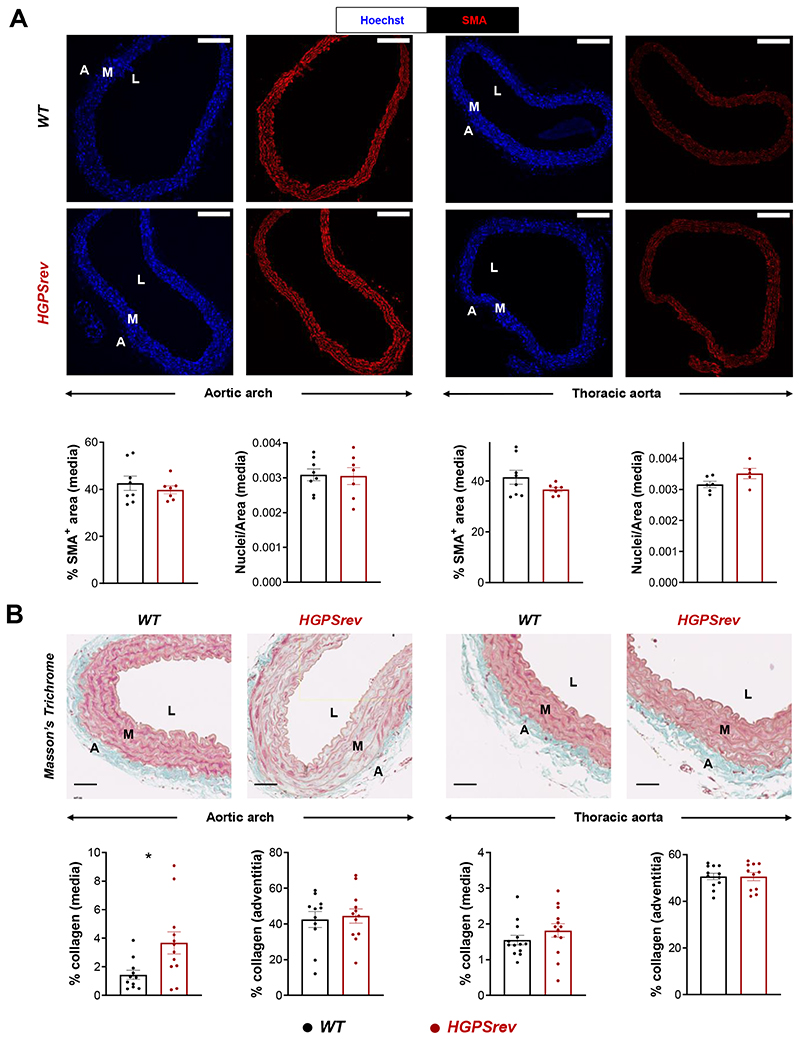
Vascular smooth muscle cell (VSMC) content and collagen deposition in the aortas of ≈8-month-old *HGPSrev* mice. **(A)** Representative immunofluorescence of cross-sections of aortic arch (*LEFT*) and thoracic aorta (*RIGHT*) stained with anti-smooth muscle α-actin (SMA) antibody (red) and Hoechst 33342 (blue) to visualize vascular smooth muscle cells (VSMCs) and nuclei, respectively. Graphs show quantification of VSMC content in the media as either the percentage (%) of SMA-positive area or nuclear density (n=6-8 *WT*; n=5-7 *HGPSrev*). Scale bar, 150 μm. **(B)** Representative images and quantification of Masson’s trichrome staining to visualize medial and adventitial collagen content in cross-sections of aortic arch (*LEFT*) and thoracic aorta (*RIGHT*) (n=11-13 *WT*; n=11-13 *HGPSrev*). Scale bar, 50 μm. Data are mean±SEM. Each symbol represents one animal. Statistical analysis was performed by two-tailed t-test (*, p<0.05). A, adventitia; L, lumen; M, media.

*HGPSrev* mice looked normal at birth and maintained a normal appearance until aged ≈5 months, when both males and females stopped gaining weight (Figure 3A). From this age, *HGPSrev* mice also exhibited kyphosis (Figure 3B) and showed a significant fat loss, as assessed from subcutaneous fat histology (Figure 3C) and *in vivo* magnetic resonance imaging (MRI) (Figure 3D). At ≈8 months of age, *HGPSrev* mice had below-normal levels of plasma low-density lipoprotein, but other lipids were normal (Figure 4A **in the Supplement**). In ≈13-month-old *HGPSrev* mice, plasma triglycerides, total cholesterol, and high-density-lipoprotein levels were reduced (Figure 4B **in the Supplement**), consistent with observations in other progerin-expressing mouse models^20^. Progerin expression was also associated with premature death in *HGPSrev* mice, which had a median lifespan of 15 months *versus* 26 months in *WT* controls (43.5% reduction) (Figure 3E).

**Figure 5:**
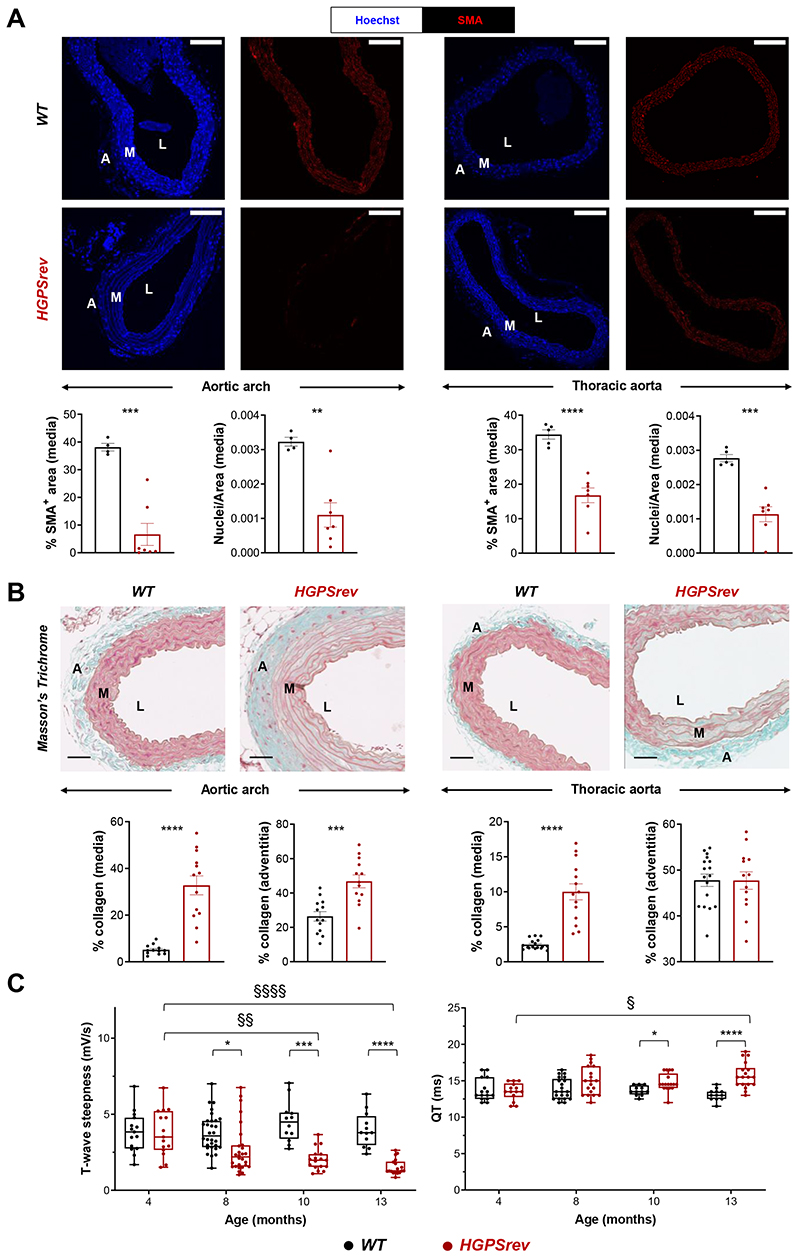
Cardiovascular abnormalities in ≈13-month-old *HGPSrev* mice. **(A)** Representative immunofluorescence images of aortic arch (*LEFT*) and thoracic aorta (*RIGHT*). Specimens were co-stained with anti-smooth muscle α-actin (SMA) antibody (red) and Hoechst 33342 (blue) to visualize vascular smooth muscle cells (VSMCs) and nuclei, respectively. Graphs show quantification of VSMC content in the media as either the percentage (%) of SMA-positive area or nuclear density (n=4-5 *WT*; n=7 *HGPSrev*). Scale bar, 150 μm. Data are mean±SEM. Statistical analysis was performed by two-tailed t-test (**, p<0.01; ***, p<0.001; ****, p<0.0001). **(B)** Representative images and quantification of Masson’s trichrome staining to visualize medial and adventitial collagen content in aortic arch (*LEFT*) and thoracic aorta (*RIGHT*) of ≈13-month-old mice (n=13-17 *WT*; n=13-14 *HGPSrev*). Scale bar, 50 μm. Data are mean±SEM. Statistical analysis was performed by two-tailed t-test (***, p<0.001; ****, p<0.0001). **(C)** Longitudinal electrocardiography (ECG) assessment (n=12-23 *WT*; n=14-19 *HGPSrev*). Data are median with interquartile range±minima and maxima. Differences were analyzed by mixed-effects analysis using the Geisser-Greenhouse correction and Sidák’s multiple comparisons test. Differences over time within each genotype: §, p<0.05; §§, p<0.01; §§§§, p<0.0001. Differences between genotypes at each timepoint: *, p<0.05; ***, p<0.001; ****, p<0.0001. Each symbol represents one animal. A, adventitia; M, media; L, lumen.

HGPS patients and animal models are both characterized by VSMC loss and collagen accumulation in the artery wall, as well as electrocardiographic alterations^7, 14, 20–30^. In ≈8-month-old *HGPSrev* mice, medial VSMC content appeared normal in both the aortic arch and thoracic aorta (Figure 4A); however, the media of the aortic arch showed increased collagen accumulation compared with age-matched *WT* mice (Figure 4B). Disease progression manifested as severe VSMC depletion in the aortic arch and thoracic aorta in ≈13-month-old *HGPSrev* mice (Figure 5A), which also had a significantly elevated collagen content in both the media and adventitia of the aortic arch and in the media of the thoracic aorta (Figure 5B). Likewise, longitudinal ECG assessment of *HGPSrev* mice revealed an age-dependent reduction in T-wave steepness from 8 months of age and an increase in the QT interval from 10 months (Figure 5C). These findings demonstrate that *HGPSrev* mice, like HGPS patients, appear healthy at birth and progressively develop the main features of the human disease, including cardiovascular alterations and premature death.

### Ubiquitous progerin suppression and lamin A restoration extends lifespan when induced in mildly symptomatic and in severely ill *HGPSrev* mice

**Figure 6:**
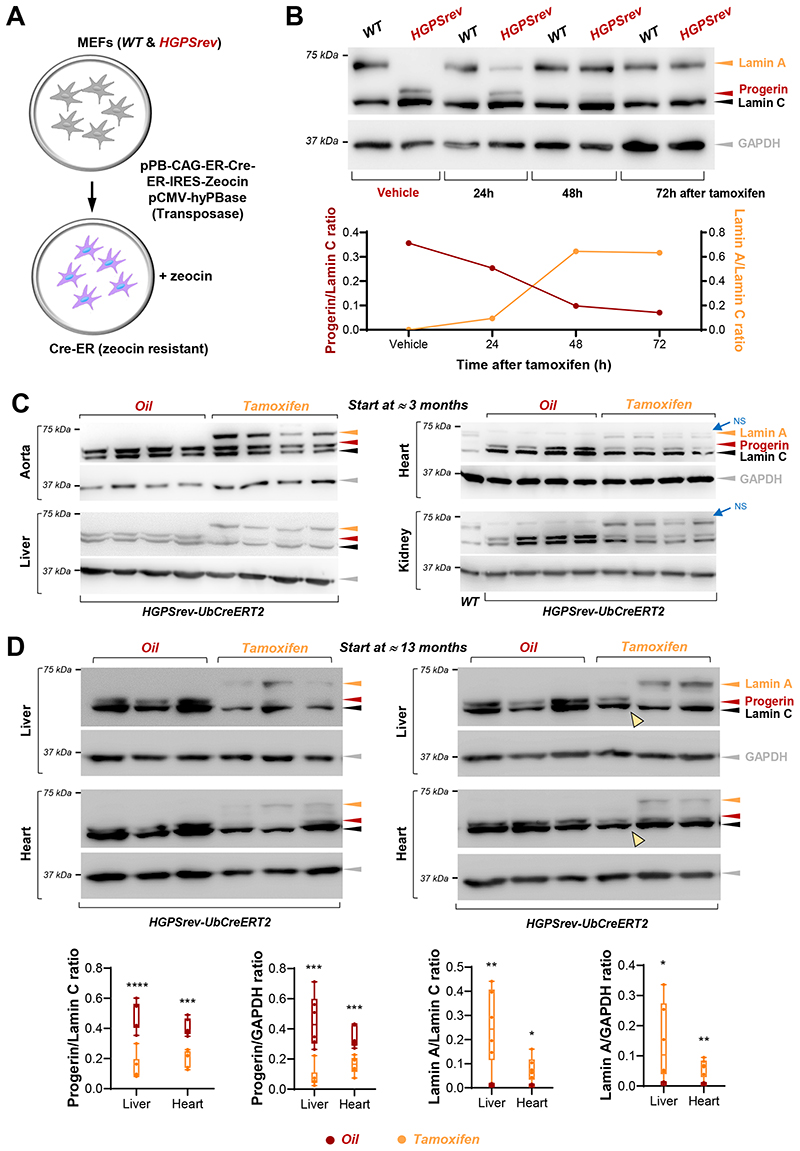
*In vitro* and *in vivo* tamoxifen-induced Cre-dependent progerin suppression and lamin A restoration. **(A)** *Wild-type* (*WT*) and *HGPSrev* mouse embryonic fibroblasts (MEFs) were co-transfected with plasmids to confer resistance to zeocin and express a tamoxifen-inducible Cre recombinase. **(B)** Zeocin-resistant MEFs were analyzed by western blot to examine lamin A/C, progerin and GAPDH expression. Equal volumes of ethanol or tamoxifen (25 nM final concentration) were added to the cells as indicated. The graph shows the relative amount of progerin and lamin A in *HGPSrev* MEFs (normalized to lamin C content). **(C, D)** Western blot analysis of tissues of *Lmna^HGPSrev/HGPSrev^ Ubc-CreER^T2-tg/+^* mice which received vehicle (oil) or tamoxifen beginning at the age of ≈3 months (C, n=4 each group) and ≈13 months (D, n=6 each group). Mice in C were euthanized 1 week after oil or tamoxifen administration, and mice in D when they met human end-point criteria. Yellow arrowheads in D indicate one animal in which tamoxifen administration did not suppress progerin or induce lamin A and that died 2 days after the end of tamoxifen administration (**see** Figure 7B, **bottom right**). Quantification of the relative amounts of lamin A and progerin in the blots in C is shown in Supplemental Figure S5). The graphs in D show the relative amount of progerin and lamin A normalized to lamin C and GAPDH content. Statistical analysis to compare genotypes was performed by two-tailed t-test. *, p<0.05; **, p<0.01; ***, p<0.001; ****, p<0.0001. Each symbol represents 1 animal. NS in panel C indicates nonspecific band.

Treatment of HGPS patients has been initiated at widely differing ages and disease stages^6^, but how late treatment can be started and still ameliorate symptoms remains unknown. The *HGPSrev* model was designed to address this key question by taking advantage of Cre recombinase expression to remove the progerin-expressing cassette and restore lamin A expression (Figure 1A). We first performed *in vitro* studies in *WT* and *HGPSrev* MEFs that were stably transfected with a vector encoding a tamoxifen-inducible Cre recombinase and a zeocin-resistance cassette (Figure 6A). Western blot assays of zeocin-resistant cells showed complete progerin suppression and lamin A restoration in *HGPSrev* MEFs 72 hours after tamoxifen administration, with no effects in tamoxifen-treated *WT* MEFs (Figure 6B). To test the system *in vivo,* we generated *HGPSrev-Ubc-CreER^T2^* mice by crossing *HGPSrev* mice with transgenic *Ubc-CreER^T2-tg/+^* mice, which ubiquitously express a tamoxifen-inducible Cre recombinase^13^. Compared with age-matched oil (vehicle)-injected controls, ≈3-month-old *HGPSrev-Ubc-CreER^T2^* mice injected with tamoxifen and sacrificed 1 week later displayed progerin downregulation and lamin A expression in all tissues tested (Figure 6C, and quantification in Figure 5 **in the Supplement**). Tamoxifen injection in ≈13-month-old *HGPSrev-Ubc-CreER^T2^* mice also resulted in progerin downregulation and lamin A restoration (Figure 6D).

**Figure 7:**
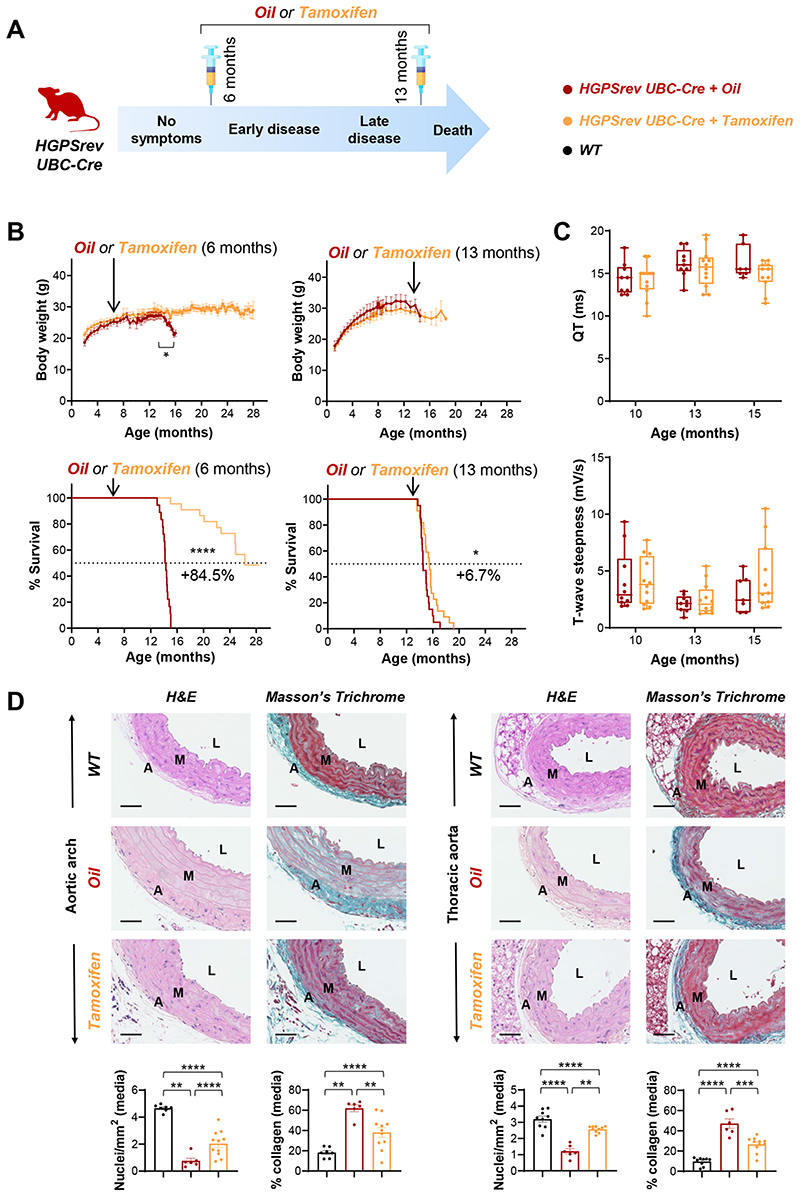
Ubiquitous progerin suppression and lamin A restoration extends lifespan in both mildly and severely symptomatic *HGPSrev-Ubc-CreER^T2^* mice. **(A)** Experimental protocol for studies with *HGPSrev-Ubc-CreER^T2^* mice, showing the age at which oil or tamoxifen administration started (**details in** Table 1). **(B)** Oil or tamoxifen were administered at ≈6 months (*LEFT:* n=18 Oil and n=22 Tamoxifen) or ≈13 months (*RIGHT:* n=7-20 Oil and n=9-23 Tamoxifen). The graphs show the results from two independent experiments. Differences were analyzed by unpaired multiple t-tests and the Holm-Sidák correction in body-weight studies and by the Mantel-Cox test in Kaplan-Meier survival curves. **(C)** Oil or tamoxifen were administered at ≈9 months of age, and electrocardiography was performed at the indicated ages (n=7-10 Oil; n=11-12 Tamoxifen). Data are medians with interquartile range±minima and maxima. Differences were analyzed by mixed-effects analysis with the Geisser-Greenhouse correction and Sidák’s multiple comparisons test. **(D)** Hematoxylin & eosin (H&E) and Masson’s trichrome staining of aortic cross-sections from mice receiving oil/tamoxifen at ≈9 months and sacrificed at 14.5 months of age (n=5-6 Oil; n=11 Tamoxifen). A group of age-matched untreated *WT* mice was included for comparison (n=7-9). Scale bar, 50 μm. Data are mean±SEM. Differences were analyzed by one-way ANOVA and the post-hoc Tukey test. **, p<0.01; ***, p<0.001; ****, p<0.0001. Each symbol represents one animal. A, adventitia; L, lumen; M, media.

To assess the impact of *in vivo* systemic progerin suppression and lamin A restoration starting at different stages of HGPS progression, we defined two ages for the start of tamoxifen administration, representing early disease (≈6 months: beginning of growth failure, one of the earliest symptoms in HGPS patients) and late disease (≈13 months: close to maximum survival) (Figure 7A **and** Table 1). Treating ≈6-month-old *HGPSrev-Ubc-CreER^T2^* mice with tamoxifen prevented the body-weight loss observed in oil-injected controls at late disease stages (Figure 7B, **top left graph**), but body weight remained below *WT* values in mice older than 12 months (see *WT* in Figure 3A). Despite this, progeroid mice injected with tamoxifen at ≈6 months showed an 84.5% increase in median lifespan (p<0.0001 versus oil; experiment ongoing at the time of manuscript submission: all oil-treated controls had died by ≈15 months of age, whereas 13 out of 22 tamoxifen-treated mice were still alive and in good health at ≈27 months of age) (Figure 7B, **bottom left graph**). Beginning tamoxifen administration in ≈13-month-old *HGPSrev-Ubc-CreER^T2^* mice with severe symptoms resulted in progerin downregulation and lamin A expression (Figure 6D), and increased median lifespan by 6.7% (p<0.05 versus oil-treated mice) (Figure 7B, **bottom right graph**). These results demonstrate that administering tamoxifen to *HGPSrev-Ubc-CreER^T2^* mice even at very advanced disease stages significantly increases lifespan, although the benefit is much more pronounced when progerin suppression and lamin A restoration are achieved in mildly symptomatic mice.

We next investigated how suppressing progerin and restoring lamin A expression affects the cardiovascular phenotype of *HGPSrev-Ubc-CreER^T2^* mice. Tamoxifen was administered to *HGPSrev* mice at ≈9 months of age (Table 1), an intermediate disease stage when the mice already showed a clear reduction in body weight (Figure 3A) and had begun to develop cardiovascular alterations (Figure 4B **and** Figure 5C). Mice were sacrificed at ≈14.5 months of age (at which stage 4 out of 10 oil-injected animals but only 1 out of 12 tamoxifen-injected animals had died). Longitudinal studies showed no statistically-significant differences in ECG parameters between both groups of mice at all ages tested (Figure 7C). In contrast, histological analysis revealed increased VSMC number and decreased medial collagen content in the aortic arch and thoracic aorta of tamoxifen-injected *HGPSrev-Ubc-CreER^T2^* mice relative to oil-injected controls, although tamoxifen did not normalize these parameters to the values seen in *WT* mice (Figure 7D).

### Progerin suppression and lamin A restoration in vascular smooth muscle cells and cardiomyocytes is sufficient to prevent vascular damage and to normalize lifespan in *HGPSrev* mice

**Figure 8:**
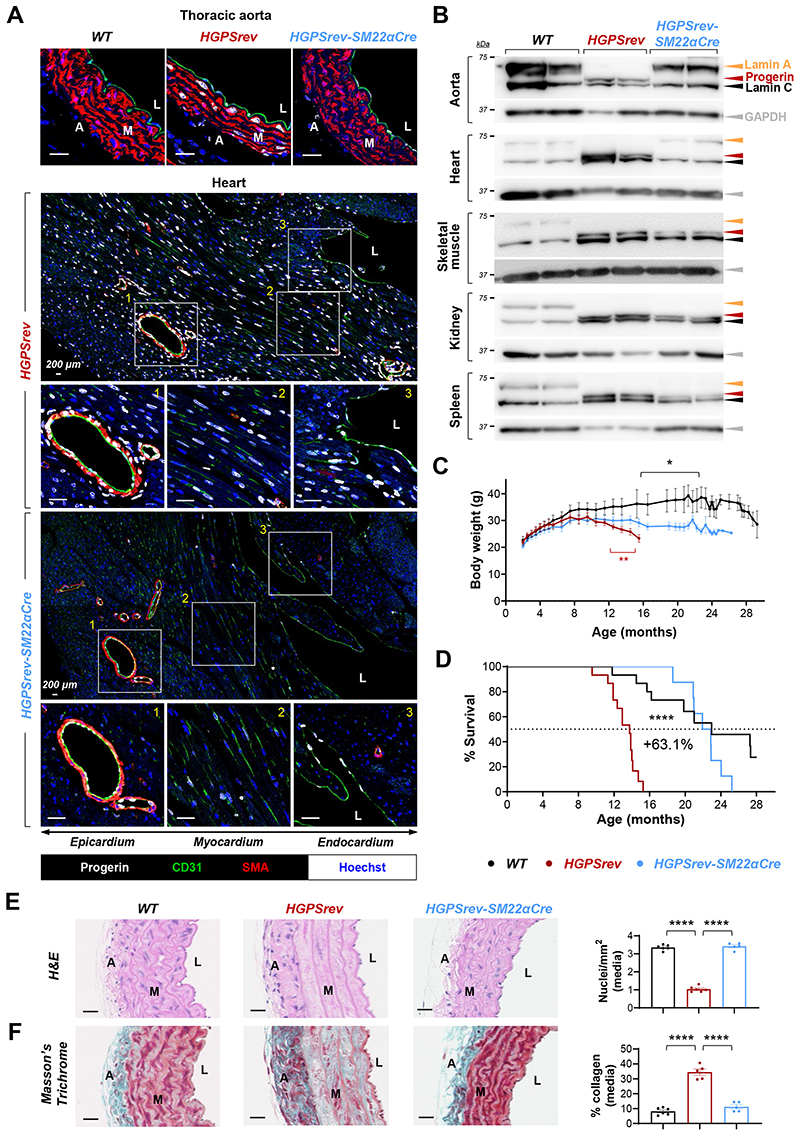
Normal vascular phenotype and lifespan in *HGPSrev-SM22α-Cre* mice with progerin suppression and lamin A restoration restricted to vascular smooth muscle cells (VSMCs) and cardiomyocytes. **(A)** Representative immunofluorescence images of thoracic aorta and hearts of ≈l3-month-old mice. Cross-sections were co-stained with antibodies against CD31 (green), smooth muscle α-actin (SMA) (red) and progerin (white) and with Hoechst 33342 (blue) to visualize endothelial cells, vascular smooth muscle cells, progerin, and nuclei, respectively. **(B)** Western blot of lamin A/C, progerin and GAPDH in tissues of ≈l3-month-old mice. **(C)** Body-weight curves (n=9 *WT*; n=13 *HGPSrev,* n=11 *HGPSrev-SM22α-Cre*). Differences were analyzed by unpaired multiple t-tests and the Holm-Sídák correction. Red asterisks denote differences between *HGPSrev-SM22α-Cre* and *HGPSrev* mice. Black asterisks denote differences between *HGPSrev-SM22α-Cre* and *WT* mice. **(D)** Kaplan-Meier survival curve (n=15 *WT*; n=15 *HGPSrev*; n=11 *HGPSrev-SM22α-Cre*). Median lifespan was 13.73 months in *HGPSrev* mice, 22.4 months in *HGPSrev-SM22α-Cre* mice, and 22.97 months in *WT* mice. Differences were analyzed with the Mantel-Cox test. **(E, F)** Representative images of aortic arch stained with hematoxylin & eosin (H&E) and Masson’s trichrome, to quantify vascular smooth muscle cells (VSMCs) and fibrosis, respectively, in ≈l3-month-old *WT* mice (n=6), *HGPSrev* mice (n=5-6), and *HGPSrev-SM22α-Cre* mice (n=5). Differences were analyzed by one-way ANOVA with the post-hoc Tukey test. *, p<0.05; ****, p<0.0001. Each symbol represents one animal. Data are mean±SEM. A, adventitia; L, lumen; M, media. Scale bars, 25 μm (except the ones in the tile scans in panel A, where they account for 200 μm).

To investigate whether all affected tissues should be targeted to ameliorate disease symptoms, or whether tissue-specific therapies would be effective, we examined the effect of suppressing progerin and restoring lamin A only in VSMCs and cardiomyocytes, major progerin targets in HGPS patients and animal models^2, 6, 7, 14, 20–28^. For this analysis, we used the *SM22α-Cre* transgenic line —which allows Cre-dependent recombination in VSMCs and cardiomyocytes^31^— to generate *HGPSrev-SM22α-Cre* mice. Immunofluorescence experiments confirmed that progerin expression was undetectable in arterial VSMCs of *HGPSrev-SM22α-Cre* mice and was clearly reduced in non-endothelial cardiac cells (69±3.8% progerin-positive cells in *HGPSrev* mice *versus* 17±2.8% in *HGPSrev-SM22α-Cre* mice; p<0.0001 by unpaired two-tailed Student t-test), but remained robust in endothelial cells in aorta and heart (Figure 8A) and in non-cardiovascular tissues (Figure 2 **in the Supplement**). Progerin elimination and lamin A restoration were also confirmed by western blot assays in *HGPSrev-SM22α-Cre* aorta and heart, whereas progerin and lamin C expression and lack of lamin A were observed in skeletal muscle, kidney, and spleen (Figure 8B). Although *HGPSrev-SM22α-Cre* mice had a higher body weight than *HGPSrev* controls from 12 months of age, they were significantly thinner than age-matched *WT* mice from ≈17 months of age (Figure 8C). Despite this, the lifespan (Figure 8D), aortic medial VSMC density (Figure 8E), and collagen content (Figure 8F) of *HGPSrev-SM22α-Cre* mice were undistinguishable from those of *WT* mice. These findings complement our recent studies showing that *SM22α*-Cre-driven progerin expression is sufficient to promote cardiovascular alterations in mice^20, 29, 30, 32^ and demonstrate that targeting progerin in VSMCs and cardiomyocytes is sufficient to normalize life expectancy despite the reduced body weight caused by progerin expression in other cell types.

## Discussion

We report here the generation and characterization of *HGSrev* mice, a new HGPS model that features ubiquitous progerin and lamin C expression and lack of lamin A, and that allows Cre-dependent progerin suppression and lamin A restoration in a time- and cell-type-specific manner. *HGPSrev* mice progressively develop many symptoms characteristic of the human disease, including growth failure, lipodystrophy, VSMC loss, vascular fibrosis, electrocardiographic anomalies, and premature death. Interestingly, we found increased collagen accumulation in the aortic media of *HGPSrev* mice at ages when VSMC content still does not differ from that of *WT* mice. Likewise, previous studies in 8-week-old *Lmna^G609G/G609G^* mice that still exhibited normal VSMC content revealed alterations in genes involved in aortic fibrosis^32^ and higher collagen III and lysyl oxidase expression in carotid arteries^33^, suggesting that progerin-induced extracellular matrix alterations precede VSMC death.

Unlike HGPS patients and *Lmna^G609G/G609G^* and *LMNA G608G* transgenic mice, two widely-used HGPS models^14, 26^, *HGPSrev* mice exhibit undetectable lamin A expression; however, mice that ubiquitously express lamin C and lack lamin A appeared normal^14, 34, 35^, suggesting that lamin A absence is unlikely to provoke progeroid symptoms in *HGPSrev* mice. Indeed, *HGPSrev* mice develop disease symptoms more slowly and die later than homozygous *LMNA G608G* mice^36^ and *LMNa^G609G/G609G^* mice^14^. Our proteomic studies in *HGPSrev* and *LMNa^G609G/G609G^* mice revealed similar amounts of farnesylated progerin in both models when normalized to the total amount of A-type lamin isoforms, but *HGPSrev* mice exhibited lower progerin mRNA level in all tissues tested, which may explain, at least in part, their milder phenotype.

Because HGPS is a progressive disease and children with this condition are diagnosed after the appearance of symptoms^6^, it is critical to determine the reversibility of progerin-induced damage and the optimal time window for treatment, chief questions that remain unanswered. In progerin-expressing mice, the progeroid phenotype can be ameliorated and lifespan increased by CRISPR-Cas9^37, 38^ and base-editing approaches^36^ to ubiquitously correct the HGPS-causing mutation or by the delivery of antisense oligonucleotides to block pathogenic splicing of mutant lamin A transcripts^14, 39, 40^; however, these treatments were administered to asymptomatic neonates and young animals. We found an 84.5% extension in median lifespan when progerin was suppressed and lamin A restored in mildly symptomatic ≈6-month-old *HGPSrev* mice. This benefit in survival occurred despite the below-normal body weight of these animals, consistent with previous mouse studies and clinical trials demonstrating lifespan extension without bodyweight normalization after the administration of various treatments (e.g., ^3, 8, 14, 32, 36–40^). Future studies are warranted to test whether early disease stages feature irreversible adipocyte death that causes persistent lipodystrophy. Importantly, progerin suppression and lamin A restoration also prolonged lifespan in severely affected progeroid *HGPSrev* mice. Although phenotypic amelioration was much more pronounced when progerin suppression and lamin A restoration were achieved in early disease stages, these results strongly suggest that it is never too late to start treatment for HGPS. Indeed, tipifarnib has been found to prevent the late progression of existing cardiovascular defects when administered to progeroid mice with overt disease symptoms^41^. Moreover, lonafarnib improved carotid-femoral pulse wave velocity and other outcome measures in HGPS clinical-trial participants who started treatment at advanced disease stages^8^ and also prolonged survival in a trial population with an average age of enrollment of 8.4 years^3^.

In a background of normal expression of endogenous wild-type *Lmna*, Eriksson's laboratory generated transgenic mice with doxycycline-inducible expression of progerin in skin or osteoblasts/odontoblasts, which showed skin or bone/teeth defects upon doxycycline administration, respectively^42, 43^. Remarkably, the defects in these mutant mice were normalized by turning off progerin expression after the appearance of the phenotype. These seminal studies in mice demonstrated that ectopic progerin expression in skin and bone does not cause irreversible damage to these tissues in the context of normal endogenous lamin A expression. Nonetheless, it is important to note that HGPS patients express below-normal levels of lamin A and that progerin is expressed in a broad range of tissues. Moreover, a major medical problem in HGPS is severe cardiovascular disease. Thus, uncertainty remained about the ability of progerin suppression and lamin A restoration to halt disease progression and increase lifespan when administered only in cardiovascular cells, the major progerin targets. We found that vascular abnormalities and premature death are both prevented in *HGPSrev-SM22α-Cre* mice with progerin suppression and lamin A restoration restricted to VSMCs and cardiomyocytes. This benefit occurred despite sustained broad progerin expression in other cell types, which was associated with significantly reduced body weight compared with age-matched *WT* mice. Although our model does not differentiate between effects in VSMCs and cardiomyocytes, we found that lifespan extension after ubiquitous progerin suppression and lamin A restoration in symptomatic progeroid mice was associated with reductions in both VSMC loss and collagen accumulation in the aortic media but did not ameliorate electrocardiographic defects. Moreover, HGPS is not known to be a cardiomyopathy, and massive VSMC loss and accumulation of extracellular matrix components have been observed in the arterial wall of HGPS mouse models and patients^7, 14, 20, 23, 25–30^. Taken together, these findings suggest a major role of vascular disease in HGPS. Further studies using the *HGPSrev* model and Cre transgenic lines specifically targeting VSMCs or cardiomyocytes will conclusively show the relative contribution of these cell types to disease progression and premature death, which may help optimize gene therapy or RNA therapy to treat HGPS.

## Supplementary Material

Supplemental Publication Material

## Figures and Tables

**Table 1:** Age of *Lmna^HGPSrev^-Ubc-CreER^T2^* mice at the time of initiation of oil or tamoxifen administration

Disease stage (age initiation oil/tamoxifen administration)	Age at initiation of oil/tamoxifen administration (mean ± SD)	Experiments
Early disease (≈6-month-old; symptoms emerging)	24.3 ± 2.15 weeks	Body-weight evolution and survival
Intermediate disease (≈9-month-old; mild symptoms)	35.9 ± 3.2 weeks	Electrocardiography and histopathology
Late disease (≈13-month-old; severe symptoms)	54.4 ± 1.97 weeks	Body-weight evolution and survival

SD, standard deviation

## Clinical Perspective

### What is new?

- We have generated a new HGPS-like mouse model that ubiquitously expresses progerin, lacks lamin A, and allows progerin suppression and lamin A restoration in a time- and cell-type-specific manner upon Cre recombinase activation.
- Progerin suppression and lamin A restoration extended lifespan in mice with mild symptoms and even in severely ill animals, although the benefit was much more apparent in mildly symptomatic animals.
- Despite the broad expression pattern of progerin and its deleterious effects in many organs, restricting its suppression and lamin A restoration to VSMCs and cardiomyocytes prevented the development of vascular pathology and normalized lifespan in *HGPSrev* mice.

### What are the clinical implications?

- Our preclinical studies demonstrate that it is never too late to treat HGPS, although the benefit was much more pronounced when progerin suppression and lamin A restoration were achieved at early stages of disease progression.
- Strategies to treat HGPS through gene therapy or RNA therapy should consider targeting VSMCs and cardiomyocytes.
