# Supplementary material for: Cardiovascular progerin suppression and lamin A restoration rescues Hutchinson-Gilford progeria syndrome
Source: Circulation. Author manuscript; Available in PMC 2021 Nov 30. (PMC8614561; doi:10.1161/CIRCULATIONAHA.121.055313)
Supplement: Supplemental Publication Material [file EMS137418-supplement-Supplemental_Publication_Material.pdf]

SUPPLEMENTAL MATERIAL

Cardiovascular progerin suppression and lamin A restoration rescues Hutchinson-Gilford progeria syndrome

Amanda Sánchez-López PhD[1,2,†], Carla Espinós-Estévez MS[1,†], Cristina González-Gómez BS[1,2], Pilar Gonzalo PhD[1,2], María J. Andrés-Manzano[1,2], Víctor Fanjul PhD[1,2], Raquel Riquelme-Borja MS[1], Magda R. Hamczyk PhD[1,2,††], Álvaro Macías PhD[1,2], Lara del Campo PhD[1,2,†††], Emilio Camafeita PhD[1,2], Jesús Vázquez PhD[1,2], Anna Barkaway PhD[3], Loïc Rolas PhD[3], Sussan Nourshargh PhD[3], Beatriz Dorado PhD[1,2], Ignacio Benedicto PhD[1], Vicente Andrés PhD[1,2,*]

[1] Centro Nacional de Investigaciones Cardiovasculares Carlos III (CNIC), Madrid, Spain.
[2] CIBER de Enfermedades Cardiovasculares (CIBERCV), Spain.
[3] Centre for Microvascular Research, William Harvey Research Institute, Barts and The London School of Medicine and Dentistry, Queen Mary University of London, London, UK.

[†] Co-first authors with equal contribution

[††] Current address: Departamento de Bioquímica y Biología Molecular, Instituto Universitario de Oncología (IUOPA), Universidad de Oviedo, 33006 Oviedo, Spain

[†††] Current Address: Departamento de Biología Celular, Facultad de Medicina, Universidad Complutense de Madrid, 28040 Madrid, Spain

* Corresponding author:
Vicente Andrés
CNIC, Melchor Fernández Almagro 3, 28029 Madrid (Spain)
Phone: +34-91 453 12 00 (Ext. 1502)
E-mail: vandres@cnic.es

CONTENT

- **Supplemental Methods**

- **Table S1:** Microinjection solution injected into zygotes and crRNA and tracrRNA sequences used.

- **Table S2:** Primer sequences used to identify founder *Lmna*^{HGPSrev} mice and for genotyping *Lmna*^{HGPSrev} mice and *Lmna*^{HGPSrev}*-Ubc-CreER*^{T2} mice.

- **Figure S1:** Double-stranded DNA donor template sequence used to generate the *Lmna*$^{HGPSrev/HGPSrev}$ *(HGPSrev)* mouse model and analysis of lamin A/C and progerin expression.

- **Figure S2.** Progerin expression in *wild-type (WT), Lmna*$^{HGPSrev/HGPSrev}$ *(HGPSrev),* and *HGPSrev-SM22α-Cre* mice.

- **Figure S3:** Analysis of potential off-target effects in *Lmna*$^{HGPSrev/HGPSrev}$ *(HGPSrev)* mice.

- **Figure S4:** Plasma lipid concentrations in wild-type *(WT)* and *Lmna*$^{HGPSrev/HGPSrev}$ *(HGPSrev)* mice.

- **Figure S5:** Quantification of lamin A and progerin expression in tissues of *HGPSrev-Ubc-CreER*^{T2} mice treated with oil or tamoxifen.

.

SUPPLEMENTAL METHODS

Analysis of potential off-target effects of CRISPR/Cas9 in *HGPSrev* mice

To examine potential off-target (OT) effects in *HGPSrev* mice, we used the online Off-Spotter tool (https://cm.jefferson.edu/Off-Spotter/). Taking as a reference the sequence of the 20-mer sgRNA that we used for Crispr/Cas9-dependent editing, this analysis identified 184 mouse genomic sequences containing 3, 4, or 5 mismatches (2, 16, and 166 sequences, respectively) **(Figure S3A in the Supplement)**. For off-target assessment, we chose the 3-mismatch sequences (OT-1 and OT-2) and 6 of the 4-mismatch sequences (OT-3, OT-4, OT-5, OT-6, OT-7, and OT-8) **(Figure S3B in the Supplement)**, which were amplified by PCR using as template genomic DNA extracted from the tail of *WT* and *HGPSrev* mice (n=5 per genotype). PCR products were sequenced at the Centro Nacional de Investigaciones Oncológicas (CNIO, Madrid, Spain) using the Sanger method[44].

Protein extraction and western blotting

MEFs were directly lysed in cell-culture plates by scraping in SDS lysis buffer (4% SDS, 20% glycerol, and 120 mM Tris-HCl, pH 6.8). Extracts were incubated for 5 min at 95ºC and then passed 10 times through a 25-gauge needle. Protein concentration was determined from absorbance at 280 nm measured in a NanoDrop ND-1000 spectrophotometer (Nanodrop, Wilmington, DE USA).

Mouse tissues were collected, snap-frozen and preserved at -80ºC until further use. To obtain representative samples, frozen tissues were powdered and subsequently homogenized with a TissueLyser (Qiagen) in lysis buffer (50 mM Tris HCK pH 8.8, 2% SDS, 8 M Urea, 2 M thiourea). Lysates were sonicated in a Bioruptor Sonication System (Diagenode, Belgium) coupled to a Neslab RTE 7 Circulating Chiller (Thermo Fisher) for 10 min, lysed on a rotating wheel for 30-60 min at 4ºC (SB3 rotator; Stuart, Staffordshire, UK), and centrifuged at 16,000 g and 4ºC for 15 min. Protein concentration was determined by the Bradford method[45], and equal amounts of protein were separated by SDS-PAGE and transferred to methanol activated Immobilon-FL polyvinylidene fluoride membranes (IPVH00010; Millipore, Burlington, MA USA) using a wet transfer system (Mini Trans-Blot® Cell; Bio-Rad, Hercules, CA USA). Membranes were blocked for 60 min with 5% bovine serum albumin (BSA) in Tris-buffered saline supplemented with 0.2% Tween 20 (TBS-T), followed by an overnight incubation at 4ºC with anti-lamin A/C (1:1,500 SC-376248; Santa Cruz Biotechnology, Dallas, TX USA) and anti-GAPDH (1:15,000 MAB374, Millipore) diluted in TBS-T containing 5% BSA. After washing, membranes were incubated for 60 min at room temperature with

horseradish peroxidase (HRP)-conjugated secondary antibody (SC-516102, Santa Cruz Biotechnology; 1:10,000 for GAPDH and 1:4,000 for lamin A/C) diluted in TBS-T, 5% BSA. Blots were incubated with Luminata Forte chemiluminescent HRP substrate (Millipore) and imaged using an ImagenQuant LAS 4000 mini system (GE Healthcare, Chicago, IL USA). Relative protein amounts were quantified with Image Studio Lite (LI-COR Biosciences, Lincoln, NE USA).

Proteomics analysis for the relative quantitation of farnesylated progerin

Protein extraction – Frozen mouse hearts were powdered and processed for protein lysate preparation as described in the previous section but with lysis with RIPA buffer (50 mM Tris-HCl, 1% Triton X-100, 150 mM NaCl, 1 mM DTT, 0.1% SDS, 2 mM EDTA, 0.5% sodium deoxycholate). For immunoprecipitation, three lysates were pooled per genotype.

Immunoprecipitation – 6 µg of anti-lamin A/C antibody (SC-376248; Santa Cruz Biotechnology) were used to generate antibody-Protein G conjugates (Dynabeads™ Protein G; Invitrogen). Protein extracts (450 µL; total protein: 675-720 µg) were added to Eppendorf tubes containing anti-lamin A/C-Dynabeads™, and mixtures were incubated for 2h at 4ºC with rotation. Immunocomplexes were washed 6 times with an excess of PBS using a DynaMag™ Magnet (Invitrogen) to retain the anti-lamin A/C-Dynabead-antigen complexes in the tube and allow removal of the supernatant in each wash. The washed material was resuspended in 45 µL of PBS.

To assess specificity, controls included tubes containing only beads + antibody (CT1) and only beads + protein extract (CT2). As additional controls, we collected the supernatants directly after incubation with anti-lamin A/C-Dynabeads™ (from tubes with the DynaMag™ Magnet but before starting the washes).

Western blot – Samples were examined by western blot using the anti-lamin A/C antibody as described in the previous section.

Protein digestion – The dried anti-lamin A/C-Dynabead™-antigen complexes (approximately 80% of the post-immunoprecipitation volume) were resuspended in sample buffer (5% SDS, 10% glycerol, 25 mM Tris-HCl, pH 6.8, 10 mM dithiotreitol, 0.01% bromophenol blue) and boiled for 5 min to elute proteins. The magnetic beads were retained against the wall of the tubes with the DynaMag™ Magnet, and the supernatants were trypsin-digested separately using the one-step in-gel digestion protocol[46]. Briefly, protein solutions were loaded onto 1-cm wide wells of a conventional SDS-PAGE gel (0.5 mm-thick, 4% stacking and 10% resolving). The run was stopped when the front entered 2

mm into the resolving gel, so that the proteins became concentrated at the stacking/resolving gel interface. The unseparated protein bands were visualized by Coomassie staining, excised, cut into pieces, treated with dithiotreitol (Sigma-Aldrich) and iodoacetamide (Sigma-Aldrich), and digested overnight at 37ºC with trypsin (Promega). The resulting tryptic peptides were acidified with trifluoroacetic acid (Sigma-Aldrich), desalted in RP C-18 extraction cartridges (Oasis; Waters, Milford, MA USA), and dried.

Targeted precursor-reaction monitoring (PRM) analysis – Targeted liquid chromatography coupled to tandem mass spectrometry (LC-MS/MS) analysis of mouse heart samples was carried out by high-resolution PRM in an Ultimate 3000 nano-HPLC apparatus (Dionex, Sunnyvale, CA USA) coupled to a hybrid quadrupole-orbitrap mass spectrometer (Q Exactive HF; Thermo Scientific). Peptides were separated on a C-18 RP nanocolumn (75 µm I.D., 50 cm, Easy-spray C18; Thermo Scientific) using a 200 nL/min flow and a continuous gradient consisting of 5-45% B for 80 min and 45-90% B for 4 min (B: 90% acetonitrile, 0.1% formic acid). Each MS run consisted of enhanced FT-resolution spectra (120,000 resolution) in the 400-1200 m/z range followed by time-scheduled data-independent acquisition of MS^2 spectra from the precursor ions. Every cycle of data-independent full-MS precursor and fragment scans was repeated along the corresponding time segment. Extracted ion chromatograms of selected ion fragments were obtained with Xcalibur 2.2 (Thermo Fisher).

Magnetic resonance imaging (MRI)

MRI studies were performed at the CNIC Advanced Imaging Unit using a 7-T Agilent/Varian scanner (Agilent Technologies, Santa Clara, CA USA) equipped with a DD2 console and an actively shielded 115/60 gradient. For image acquisition, mice were sedated with 2% isoflurane in oxygen, and an ophthalmic gel was applied to prevent retinal drying. Body-fat images were acquired using a spin-echo multi-slice (SEMS) sequence with and without fat saturation and with the following parameters: 533-884 ms repetition time (varies between with and without fat saturation), 10.44 ms echo time, 128 x 128 acquisition matrix, 90º flip angle, 1 average and 35 1-mm slices with a 1 mm gap. Images were analyzed using Fiji-ImageJ software. Fat was segmented from each slice to obtain the fat volume. Fat mass was calculated as the product of fat volume and fat density (0.9 g/cm^3).

Table S1. Microinjection solution injected into zygotes and crRNA and tracrRNA sequences used.

Microinjection solution
• 0.61 μM sgRNA: 0.61 μM crRNA and 0.61 μM tracrRNA<br>• *Streptococcus pyogenes* Cas9 endonuclease, with nuclear localization signal 8NLS (PNA Bio, Cat. CP01-50)<br>• dsDNA donor template (2494 bp): 4.5 mg/μL<br>• 10x microinjection buffer: 100 mM Tris-HCl pH 7.5, 1.0 mM EDTA

	RNA sequence (5' → 3')
crRNA	CCCAUAAGUGUCUAAGAUUCGUUUUAGAGCUAUGCUGUUUUG
tracrRNA	AGCAUAGCAAGUUAAAAUAAGGCUAGUCCGUUAUCAACUUGAAAAAGUGG CACCGAGUCGGUGCUUU

Table S2. Primer sequences used to identify founder *Lmna*^{HGPSrev} mice and for genotyping *Lmna*^{HGPSrev} mice and *Lmna*^{HGPSrev}-*Ubc-CreER*^{T2} mice.

Founders	Forward (5' → 3')	Reverse (5' → 3')
**PCR-1**	CCTTCCACCCTATTGCATGC	TTCAGGCCTGCTCTCCTAAG
**PCR-2**	CATCTGTTGTTTGCCCCTCC	CTTTCCCTGGGCTCCTAGAG
**PCR-3**	AGAGTCGGTTGAACTCCCTG	CATGATGCTGCAGTTCTGGG
**Genotyping**	**Forward (5' → 3')**	**Reverse (5' → 3')**
***Lmna*^{HGPSrev}**	TCTTCCTCTTGATTGCCCCA	TAGCCAGGAAGCCTTCGAAA
Ubc-CreERT2	CGGTCGATGCAACGAGTGATGAGG	CCAGAGACGGAAATCCATCGCTCG
SM22α-Cre*	CTAGGCCACAGAATTGAAAGATCT	GTAGGTGGAAATTCTAGCATCATCC
	GCGGTCTGGCAGTAAAAACTATC	GTGAAACAGCATTGCTGTCACTT

* Genotyping was according to the protocol from The Jackson Laboratories (Bar Harbor, ME USA), available at: https://www.jax.org/Protocol/UrlAsPDF?stockNumber=017491&protocolID=22392

Figure S1 (see legend on next page)

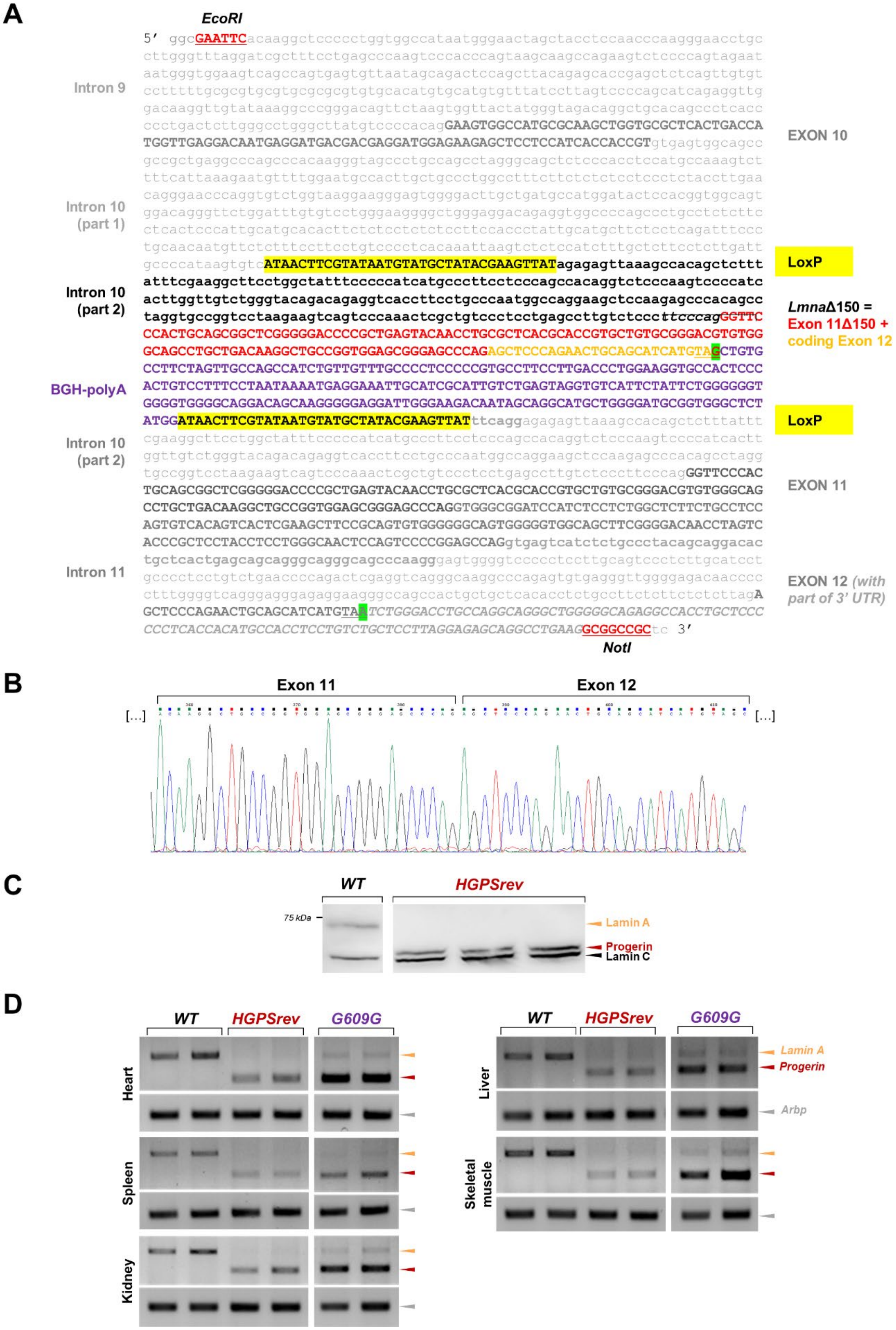

Figure S1. Double-stranded DNA donor template sequence used to generate the *Lmna*$^{HGPSrev/HGPSrev}$ *(HGPSrev)* mouse model and analysis of lamin A/C and progerin expression. (A) The reference DNA sequence used is the one available in the NCBI gene database (Gene ID: 16905, Chromosome 3, NC_000069.6, 88481148..88509932, complement). The 672-bp insert **(see Fig.1A)** contains flanking loxP sites (highlighted in yellow) and harbors part of intron 10 ('part 2', black bold typeface), exon 11 lacking the last 150 bp (bold red typeface), exon 12 (orange bold typeface), and a transcription stop signal (BGH-poly A sequence, purple bold typeface). The left (938 bp) and right (877 bp) homology arms are shown in light gray. Underlined red upper case letters show the *EcoRI* (5´) and *NotI* (3´) restriction enzyme sites used to clone the whole sequence into the pcDNA3.1 expression vector (GenScript). Green-highlighted typeface shows single base changes introduced to avoid the possible generation of alternative splice sites. **(B)** Representative genomic sequencing of the four *HGPSrev* founder mice that were generated, showing the fusion between exon 11Δ150 and exon 12 without intron 11 in the *Lmna* gene. **(C)** Western blot analysis of lamin A isoforms in protein lysates from tails of founder *HGPSrev* mice and one wild-type control mouse (*WT*). **(D)** Lamin A and progerin mRNA expression in tissues of 3-month-old wild-type (*WT)*, *HGPSrev*, and *Lmna*$^{G609G/G609G}$ *(G609G)* mice analyzed by semiquantitative PCR. Six mice of each genotype were analyzed, and representative images are shown of two mice per genotype. For each individual tissue, all PCR products were run on the same agarose gel and one image of the whole gel was acquired (lanes between *HGPSrev* and *G609G* samples have been omitted). *Arbp* expression was used as an internal control. Wells in the electrophoresis gels for western blotting and PCR analysis were loaded with samples from different mice.

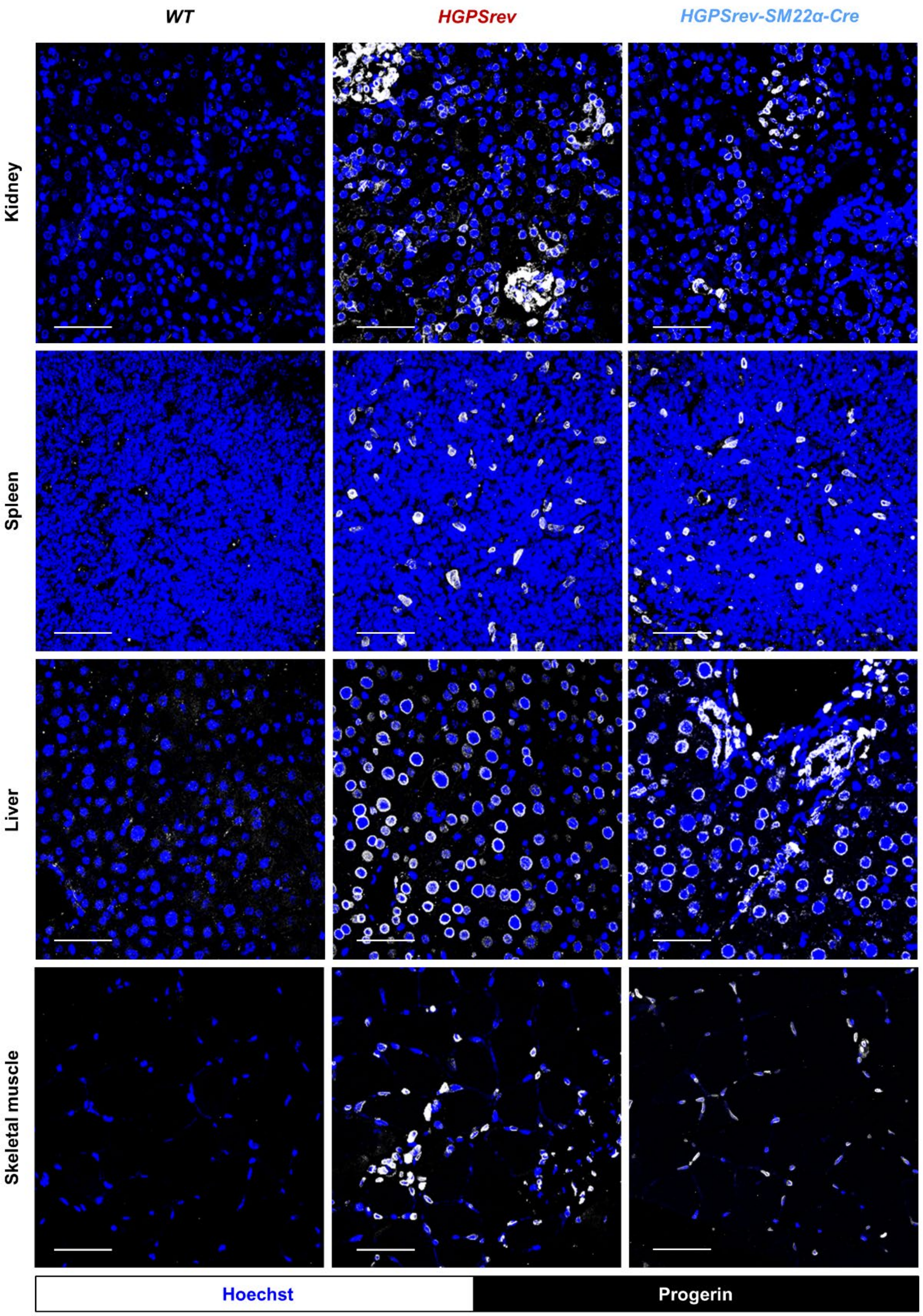

Figure S2. Progerin expression in *wild-type (WT), Lmna$^{HGPSrev/HGPSrev}$ (HGPSrev),* and *HGPSrev-SM22α-Cre* mice. Mice were sacrificed at ≈13 months of age. Representative immunofluorescence images of kidney, spleen, liver and skeletal muscle cross-sections co-stained with anti-progerin antibody (white) and Hoechst 33342 (blue) to visualize progerin and nuclei, respectively. Scale bar, 50 µm.

Figure S3 (see legend at the end of the figure)

A

20mer RNA	Number of mismatches	Number of results
CCCATAAGTGTCTAAGATTC	1	0
	2	0
	3	2
	4	16
	5	166

B

Target site[1]	DNA sequence[2]	Genomic coordinates	Primers 3'→5' [3]
OT-1	CCCATATGTGACCAAGATTCGGG	Chr11:69102215-69102237	Fwd: CTGCCGAATCAGGTAAGGAG<br>Rv: TCAGCTAGTGCCTCCCAAAC
OT-2	CCCATAACAGTTTAAGATTCAGG	Chr1:70316671-70316693	Fwd: TGCTTAACCGAGTACAAGCAC<br>Rv: GAAGAGCATTGGAATTTTAGCA
OT-3	ACCAGAAGTGTTTTAGATTCAGG	Chr13:11805628-11805650	Fwd: CACAGTGTTGACACAGGCTCA<br>Rv: CAGTTCACATGCCCACTCAT
OT-4	ACCATAAAAGTTTAAGATTCTGG	Chr7:55876988-55877010	Fwd: GGAAAGAGAACATAAAGCCGTA<br>Rv: CACTAGTACTGGAACTTCCCAGAG
OT-5	CGCAAAAGCGTGTAAGATTCTGG	Chr10:81829700-81829722	Fwd: CTCACCAAAACCCTTTGCTT<br>Rv: TGCAGCCAATCAGGGAGT
OT-6	CACATCAGTGTCTGTGATTCTGG	Chr7:14516664-14516686	Fwd: TTTCCAGGAGGAAGGGAAAC<br>Rv: CTGGTCATGATTCTGCAGTCA
OT-7	CACATATGTGTTCAAGATTCTGG	Chr4:4841473-4841495	Fwd: TCCAGACTATTGGCACAAGC<br>Rv: TGGCATGCTTAGATTTTGGA
OT-8	CCCTCCAGAGTCTAAGATTCTGG	Chr6:54936060-54936082	Fwd: TCCACGTGGTTGACTTCTGT<br>Rv: CTTTGCCGGCACTTGTTATT

C

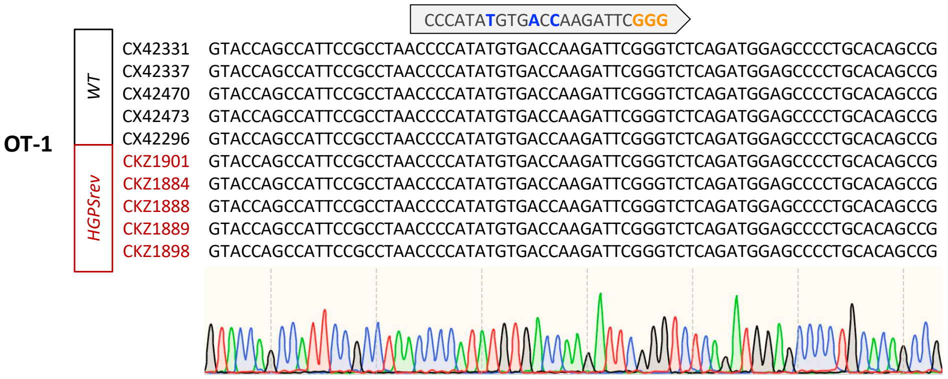

C

OT-2

CCTGAATCTTAAACTGTTATGGG

Group	Sample	Sequence
WT	CX42331	AGTTCTACTGCGTGGGGAAGCATTGCCTGAATCTTAAACTGTTATGGGATGTGAATATATTCTCACTTACA
	CX42337	AGTTCTACTGCGTGGGGAAGCATTGCCTGAATCTTAAACTGTTATGGGATGTGAATATATTCTCACTTACA
	CX42470	AGTTCTACTGCGTGGGGAAGCATTGCCTGAATCTTAAACTGTTATGGGATGTGAATATATTCTCACTTACA
	CX42473	AGTTCTACTGCGTGGGGAAGCATTGCCTGAATCTTAAACTGTTATGGGATGTGAATATATTCTCACTTACA
	CX42296	AGTTCTACTGCGTGGGGAAGCATTGCCTGAATCTTAAACTGTTATGGGATGTGAATATATTCTCACTTACA
HGPSrev	CKZ1901	AGTTCTACTGCGTGGGGAAGCATTGCCTGAATCTTAAACTGTTATGGGATGTGAATATATTCTCACTTACA
	CKZ1884	AGTTCTACTGCGTGGGGAAGCATTGCCTGAATCTTAAACTGTTATGGGATGTGAATATATTCTCACTTACA
	CKZ1888	AGTTCTACTGCGTGGGGAAGCATTGCCTGAATCTTAAACTGTTATGGGATGTGAATATATTCTCACTTACA
	CKZ1889	AGTTCTACTGCGTGGGGAAGCATTGCCTGAATCTTAAACTGTTATGGGATGTGAATATATTCTCACTTACA
	CKZ1898	AGTTCTACTGCGTGGGGAAGCATTGCCTGAATCTTAAACTGTTATGGGATGTGAATATATTCTCACTTACA

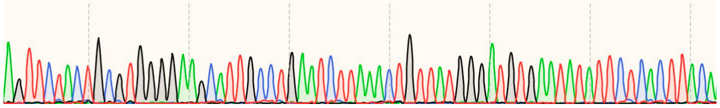

OT-3

ACCAGAAGTGTTTTAGATTCAGG

Group	Sample	Sequence
WT	CX42331	CCCCTTGCTTGCAACATCAGGACCAGAAGTGTTTTAGATTCAGGAGGTTTCCTCATTGCTGTTTTAGATTG
	CX42337	CCCCTTGCTTGCAACATCAGGACCAGAAGTGTTTTAGATTCAGGAGGTTTCCTCATTGCTGTTTTAGATTG
	CX42470	CCCCTTGCTTGCAACATCAGGACCAGAAGTGTTTTAGATTCAGGAGGTTTCCTCATTGCTGTTTTAGATTG
	CX42473	CCCCTTGCTTGCAACATCAGGACCAGAAGTGTTTTAGATTCAGGAGGTTTCCTCATTGCTGTTTTAGATTG
	CX42296	CCCCTTGCTTGCAACATCAGGACCAGAAGTGTTTTAGATTCAGGAGGTTTCCTCATTGCTGTTTTAGATTG
HGPSrev	CKZ1901	CCCCTTGCTTGCAACATCAGGACCAGAAGTGTTTTAGATTCAGGAGGTTTCCTCATTGCTGTTTTAGATTG
	CKZ1884	CCCCTTGCTTGCAACATCAGGACCAGAAGTGTTTTAGATTCAGGAGGTTTCCTCATTGCTGTTTTAGATTG
	CKZ1888	CCCCTTGCTTGCAACATCAGGACCAGAAGTGTTTTAGATTCAGGAGGTTTCCTCATTGCTGTTTTAGATTG
	CKZ1889	CCCCTTGCTTGCAACATCAGGACCAGAAGTGTTTTAGATTCAGGAGGTTTCCTCATTGCTGTTTTAGATTG
	CKZ1898	CCCCTTGCTTGCAACATCAGGACCAGAAGTGTTTTAGATTCAGGAGGTTTCCTCATTGCTGTTTTAGATTG

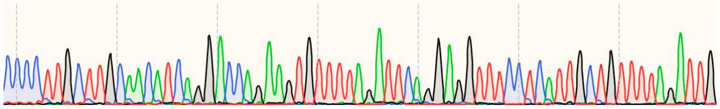

OT-4

ACCATAAAAGTTTAAGATTCTGG

Group	Sample	Sequence
WT	CX42331	GGATATGGTCCTTCCTTGCGGAACCATAAAAGTTTAAGATTCTGGGTTGCCCAGATAGAGCCTTGTGGGC
	CX42337	GGATATGGTCCTTCCTTGCGGAACCATAAAAGTTTAAGATTCTGGGTTGCCCAGATAGAGCCTTGTGGGC
	CX42470	GGATATGGTCCTTCCTTGCGGAACCATAAAAGTTTAAGATTCTGGGTTGCCCAGATAGAGCCTTGTGGGC
	CX42473	GGATATGGTCCTTCCTTGCGGAACCATAAAAGTTTAAGATTCTGGGTTGCCCAGATAGAGCCTTGTGGGC
	CX42296	GGATATGGTCCTTCCTTGCGGAACCATAAAAGTTTAAGATTCTGGGTTGCCCAGATAGAGCCTTGTGGGC
HGPSrev	CKZ1901	GGATATGGTCCTTCCTTGCGGAACCATAAAAGTTTAAGATTCTGGGTTGCCCAGATAGAGCCTTGTGGGC
	CKZ1884	GGATATGGTCCTTCCTTGCGGAACCATAAAAGTTTAAGATTCTGGGTTGCCCAGATAGAGCCTTGTGGGC
	CKZ1888	GGATATGGTCCTTCCTTGCGGAACCATAAAAGTTTAAGATTCTGGGTTGCCCAGATAGAGCCTTGTGGGC
	CKZ1889	GGATATGGTCCTTCCTTGCGGAACCATAAAAGTTTAAGATTCTGGGTTGCCCAGATAGAGCCTTGTGGGC
	CKZ1898	GGATATGGTCCTTCCTTGCGGAACCATAAAAGTTTAAGATTCTGGGTTGCCCAGATAGAGCCTTGTGGGC

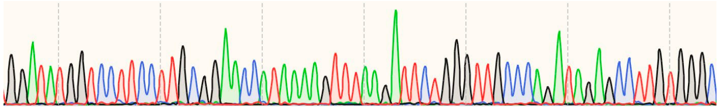

OT-5

CCAGAATCTTACACGCTTTTGCG

Group	Sample	Sequence
WT	CX42331	TGTGCATACATACCTTCCCACCCAGAATCTTACACGCTTTYGCGACCGGCCAGGAAAGACGCAACAAACC
	CX42337	TGTGCATACATACCTTCCCACCCAGAATCTTACACGCTTTYGCGACCGGCCAGGAAAGACGCAACAAACC
	CX42470	TGTGCATACATACCTTCCCACCCAGAATCTTACACGCTTTYGCGACCGGCCAGGAAAGACGCAACAAACC
	CX42473	TGTGCATACATACCTTCCCACCCAGAATCTTACACGCTTTYGCGACCGGCCAGGAAAGACGCAACAAACC
	CX42296	TGTGCATACATACCTTCCCACCCAGAATCTTACACGCTTTYGCGACCGGCCAGGAAAGACGCAACAAACC
HGPSrev	CKZ1901	TGTGCATACATACCTTCCCACCCAGAATCTTACACGCTTTYGCGACCGGCCAGGAAAGACGCAACAAACC
	CKZ1884	TGTGCATACATACCTTCCCACCCAGAATCTTACACGCTTTYGCGACCGGCCAGGAAAGACGCAACAAACC
	CKZ1888	TGTGCATACATACCTTCCCACCCAGAATCTTACACGCTTTYGCGACCGGCCAGGAAAGACGCAACAAACC
	CKZ1889	TGTGCATACATACCTTCCCACCCAGAATCTTACACGCTTTYGCGACCGGCCAGGAAAGACGCAACAAACC
	CKZ1898	TGTGCATACATACCTTCCCACCCAGAATCTTACACGCTTTYGCGACCGGCCAGGAAAGACGCAACAAACC

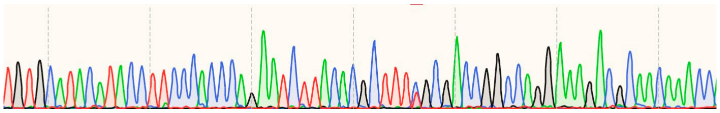

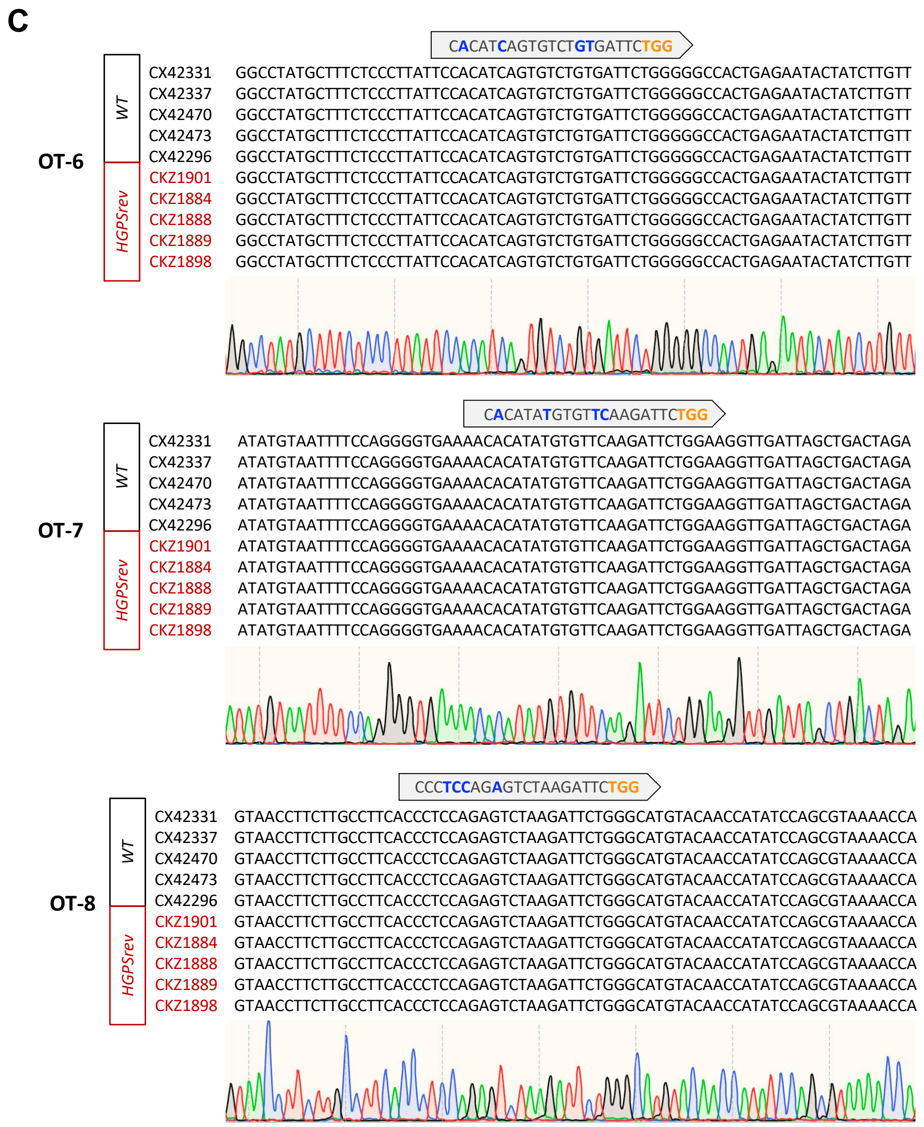

Figure S3: Analysis of potential off-target effects in *Lmna*$^{HGPSrev/HGPSrev}$ *(HGPSrev)* mice. (A) The online Off-Spotter tool was used to identify mouse genomic sequences containing up to 5 mismatches compared with the sgRNA used for Crispr/Cas9-dependent editing. The analysis identified 184 sequences containing 3, 4, and 5 mismatches (2, 16, and 166 sequences, respectively). **(B)** Sequence of the 8 potential off-target (OT) sites identified in **A** that we selected for PCR amplification of genomic DNA and sequencing, which included the 2 sequences containing 3 mismatches (OT-1, OT-2) and 6 with 4 mismatches (OT-3 to OT-8). The table shows the sequence of each OT sequence (blue: mismatches; orange: PAM sequence), their genomic coordinates, and the primers designed for PCR. **(C)** Alignment of the sequences for each OT sequence in wild-type (*WT)* and *HGPSrev* mice (n=5 per genotype). The OT sequence is shown above each alignment (color codes as in **A**). Representative chromatograms are shown below the alignments, where blue, red, green, and black correspond to C, T, A and G, respectively. The Y base in both *WT* and *HGPSrev* samples in the OT-5 analysis indicates heterozygosity at this position of C and T. Note that sgRNA can be on the positive or negative DNA strand; hence the negative strands are shown as the reverse complementary sequence.

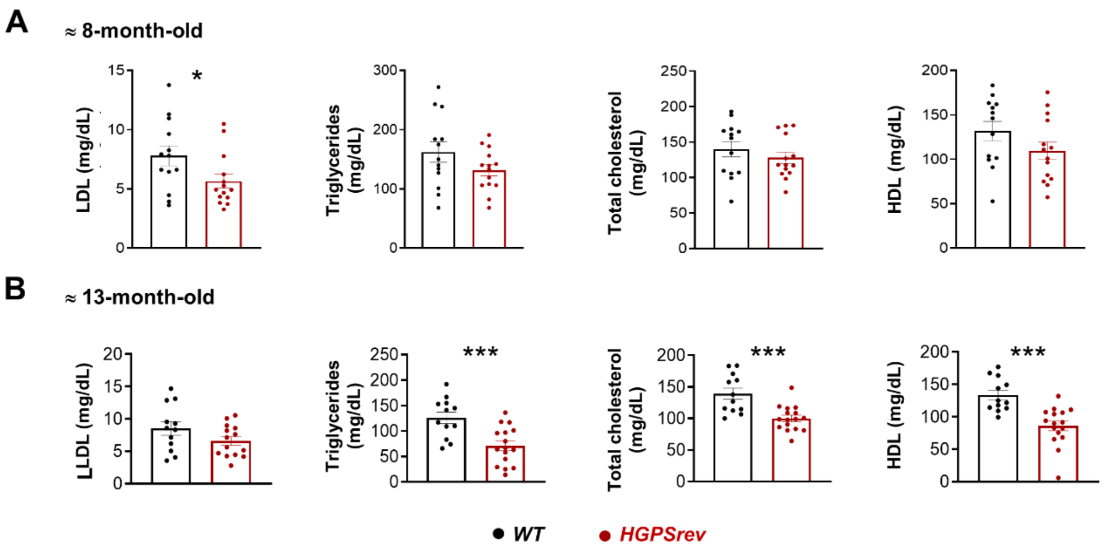

Figure S4. Plasma lipid concentrations in *wild-type (WT)* and *Lmna*$^{HGPSrev/HGPSrev}$ *(HGPSrev)* mice. (A) ≈8-month-old mice. **(B)** ≈13-month-old mice. Each symbol represents one animal. Statistical analysis was performed by two-tailed t-test. *, $p<0.05$; ***, $p<0.001$. HDL, high-density lipoprotein; LDL, low-density lipoprotein.

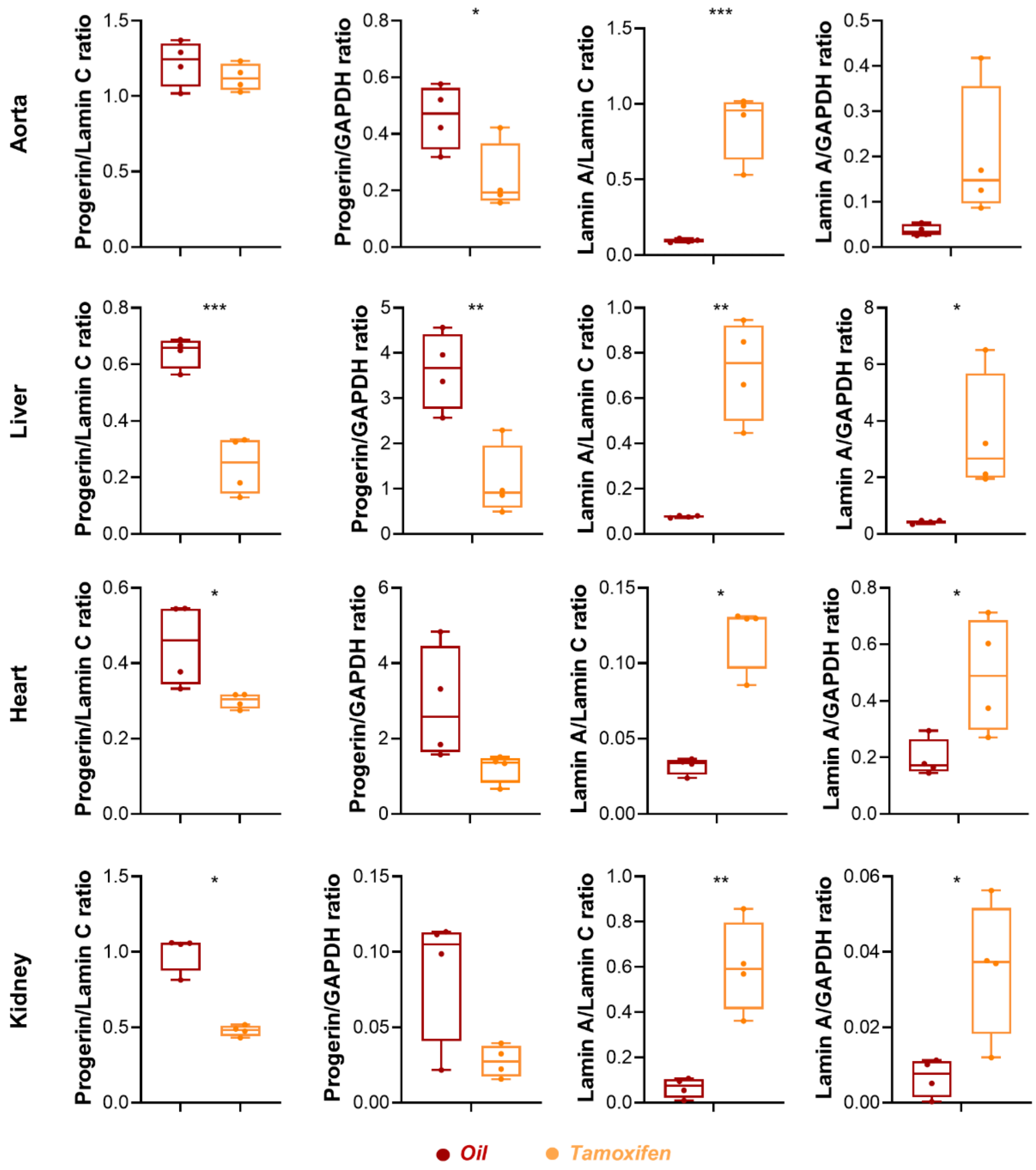

Figure S5. Quantification of lamin A and progerin expression in tissues of *HGPSrev-Ubc-CreER^{T2}* mice treated with oil and tamoxifen. The relative abundance of lamin A and progerin proteins in the western blots shown in Figure 6C was quantified using the Image Studio Lite software package (LI-COR Biosciences). Results were normalized using lamin C and GAPDH as controls (n=4 Oil-injected mice; n=4 Tamoxifen-injected mice). Statistical analysis was performed by two-tailed t-test when comparing normal populations or by Mann-Whitney test for non-normal comparisons. *, p<0.05; **, p<0.01; ***, p<0.001.
